# Edge-deployable greenhouse tomato cluster harvesting robot integrating YOLOv8n-BiFPN-WIoU and ROS-based autonomous control

**DOI:** 10.3389/fpls.2026.1926482

**Published:** 2026-10-01

**Authors:** Fanzhao Meng, Zhen Wang, Huan Wang, Xiaohui Li, Xizhen Xu, Ningrong Zeng, Guoqing Wang, Dongpo Wei, Xuan Li, Yunqi Shi

**Affiliations:** 1 School of Mechanical Engineering, Shandong Huayu University of Technology, Dezhou, China; 2 School of Advanced Manufacturing, Sun Yat-sen University, Shenzhen, China; 3 Shandong Shansu Robot Technology Co., Ltd., Dezhou, China; 4 School of Materials and Chemical Engineering, Hunan Institute of Technology, Hengyang, China

**Keywords:** agricultural robotics, cluster harvesting, edge AI, greenhouse tomato harvesting, maturity detection, precision agriculture, ROS, YOLOv8n

## Abstract

**Objective:**

To develop an edge-deployable greenhouse tomato cluster harvesting robot capable of accurate fruit detection, three-dimensional localization, autonomous navigation, and robotic harvesting under complex greenhouse conditions. The objective was to improve detection performance under fruit occlusion and variable illumination while maintaining real-time inference and stable robotic operation on embedded computing hardware.

**Methods:**

A lightweight YOLOv8n-based tomato detection model, termed YOLOv8n-BiFPN-WIoU, was developed by incorporating a bidirectional feature pyramid network and Wise-IoU loss to improve multiscale feature fusion and localization accuracy. An RGB-D vision system based on the Intel RealSense D435 was used for three-dimensional fruit localization. The robotic system integrated a rail-guided mobile platform, a six-axis manipulator, a dual-claw harvesting end-effector, and a ROS-based distributed control architecture. The vision model was optimized and deployed on an NVIDIA Jetson Nano using ONNX and TensorRT. Robot kinematics, hand-eye transformation, trajectory planning, and autonomous harvesting procedures were integrated to enable station-to-station operation and fruit harvesting.

**Results:**

The proposed YOLOv8n-BiFPN-WIoU model achieved a precision of 88.534%, recall of 89.377%, F1-score of 88.954%, mAP@0.5 of 92.338%, and mAP@0.5:0.95 of 71.368%. Compared with the baseline YOLOv8n model, these metrics improved by 2.143, 2.779, 2.460, 4.587, and 5.675 percentage points, respectively. The proposed model increased the parameter count from 3.2 million to 3.6 million and computational complexity from 19.6 to 22.4 GFLOPs. After ONNX and TensorRT optimization, the model achieved an inference speed of 21.1 frames per second at an input resolution of 640 × 640 with batch size 1 on the Jetson Nano. The robotic system achieved a mean positioning error of 3.70 ± 0.32 mm across five representative targets, demonstrating the feasibility of accurate visual localization and robotic positioning for greenhouse tomato harvesting.

**Conclusion:**

The proposed edge-deployable greenhouse tomato cluster harvesting robot integrates lightweight deep learning, RGB-D three-dimensional localization, ROS-based autonomous control, rail-guided mobile operation, and robotic harvesting into a unified system. The YOLOv8n-BiFPN-WIoU model improved tomato detection performance while maintaining real-time inference on embedded hardware, and the integrated vision and manipulation system demonstrated accurate positioning suitable for autonomous harvesting. The results indicate that the proposed architecture provides a practical foundation for intelligent greenhouse tomato harvesting under complex visual and operational conditions.

## 1 Introduction

Tomatoes are one of the most widely cultivated economic crops worldwide, with high nutritional value and strong market demand. China is the largest producer of tomatoes, with an annual output exceeding 70 million tons, accounting for more than 40% of global production. In greenhouse cultivation systems, tomatoes are typically harvested in clusters, and the harvesting timing is highly dependent on fruit ripeness. Fruits harvested too early have poor flavor and quality, while overripe fruits are prone to mechanical damage during handling and transportation. Therefore, accurately identifying optimal ripeness and performing non-destructive harvesting are essential for improving both product quality and economic returns (Goyal et al., 2024). However, current greenhouse tomato harvesting still relies heavily on manual labor, leading to high labor intensity, rising labor costs, and low operational efficiency (Rajendran et al., 2024). Additionally, greenhouse harvesting environments are characterized by dense canopy structures, severe fruit occlusion, and highly variable illumination conditions, which further increase the difficulty of accurate perception and stable robotic manipulation in real-world deployments.With the continuous expansion of greenhouse agriculture and increasing labor shortages, the development of efficient and intelligent tomato harvesting robots has become an urgent requirement.

In greenhouse environments, tomato harvesting is fundamentally a cluster-level perception and manipulation task, where multiple fruits are densely distributed on flexible stems. Unlike field harvesting, greenhouse conditions introduce strong coupling between object occlusion, non-uniform illumination, and constrained robot operation space. In recent years, extensive research has been conducted on robotic harvesting of crops such as tomatoes, apples, citrus, and strawberries (Rajendran et al., 2024). For example, vision-based detection methods using deep learning models such as You Only Look Once (YOLO) have significantly improved fruit recognition accuracy (Terven et al., 2023). ROS-based multi-sensor integration has also been employed for autonomous robotic localization and control (Irwansyah et al., 2025). YOLOv8-based models have been investigated for tomato ripeness recognition (Wang et al., 2025), while improved YOLOv8 combined with depth cameras has been used for bunch tomato stem picking-point recognition and localization (Song et al., 2024). Robotic harvesting systems have also incorporated dedicated end-effectors for automated grasping and cutting operations (Sobol et al., 2024). Real-time tomato ripeness recognition has further been demonstrated on resource-constrained computing platforms (Nahiduzzaman et al., 2025), while lightweight YOLO variants have been investigated to reduce computational requirements for agricultural target detection (Liu X. et al., 2025). Recent studies have increasingly integrated crop-specific perception, three-dimensional localization, and robotic manipulation for greenhouse tomato harvesting. Three-dimensional sensing has also been applied in other precision-agriculture tasks, such as LiDAR-based canopy profiling and canopy-volume estimation for target-specific variable spraying, demonstrating the value of spatial perception for intelligent agricultural equipment (Liu D. et al., 2025). RGB-D (red-green-blue and depth) sensing has been used to estimate tomato and peduncle positions and poses by combining color information with depth or point-cloud data, providing spatial references for robotic harvesting (Kang et al., 2025a). In particular, studies on bunch and cluster tomatoes have investigated fruit-stalk localization, picking-point detection, and observation-pose optimization under occlusion and complex greenhouse backgrounds. In parallel, compliant and pneumatic end-effectors have been developed to reduce fruit damage while maintaining reliable grasping and harvesting performance. Motion planning has also been investigated to reduce the execution time and improve trajectory efficiency of harvesting manipulators (Kang et al., 2025b). Whole-system tomato harvesting robots have also been evaluated under greenhouse or plant-factory conditions, showing that perception uncertainty, occlusion, collision, and end-effector execution remain major factors limiting harvesting success (Xu et al., 2023).In parallel, high-precision nondestructive measurement techniques based on infrared interferometry and dynamic refractive-index correction have demonstrated the value of advanced sensing and signal-processing strategies for improving measurement accuracy and robustness (Yang et al., 2026).

Improving harvesting performance and ensuring non-destructive picking are two critical objectives of harvesting robots. Fruit bruising is an important form of postharvest damage that can occur during harvesting, transportation, and subsequent handling, making damage reduction and non-destructive operation important considerations in robotic harvesting systems (Xu et al., 2026),among which the design of the end-effector and the robustness of the perception system play key roles. Existing end-effectors mainly adopt clamping, suction, or cutting mechanisms. Although soft grippers and pneumatic devices can reduce mechanical damage, they often suffer from reduced efficiency or limited adaptability to complex cluster structures. current systems typically require high positioning accuracy, increasing the complexity of control and limiting real-time performance in practical greenhouse environments. Furthermore, the coupling between perception uncertainty and mechanical execution error often leads to cumulative harvesting failures, especially in densely clustered tomato environments where multiple fruits overlap spatially. Maturity identification and category determination have also been investigated as important components of selective harvesting systems (Kang et al., 2024a).However, existing studies have rarely addressed lightweight cluster-level maturity recognition, three-dimensional localization, and low-damage harvesting execution within a unified edge-deployable robotic framework, particularly under occlusion and illumination variability in greenhouse environments.

Based on the above analysis, this study proposes a greenhouse tomato cluster harvesting robot that integrates the proposed YOLOv8n-BiFPN-WIoU visual recognition system with a ROS-based distributed control architecture. Compared with existing methods, the main contributions of this study are as follows:

1. A task-oriented enhancement strategy was developed for greenhouse tomato-cluster detection under partial occlusion. BiFPN-based bidirectional feature fusion was introduced to compensate for the loss of spatial and semantic information across feature scales when tomato clusters are partially visible, while WIoU-based regression was used to improve localization optimization for incomplete and ambiguous target boundaries. The resulting YOLOv8n-BiFPN-WIoU detector achieved an mAP@0.5 of 92.338% and an mAP@0.5:0.95 of 71.368%.
2. A complete edge-deployable greenhouse tomato cluster harvesting system was developed by integrating RGB-D-based three-dimensional target localization, ROS-based distributed control, rail-guided station-to-station motion, robotic manipulation, and a dual-claw harvesting mechanism. The proposed system establishes a complete perception-to-execution pipeline from tomato-cluster detection and depth-based localization to harvesting-pose generation and coordinated robotic execution on an NVIDIA Jetson Nano edge-computing platform (Ci et al., 2024; Weng et al., 2025; Zhang et al., 2026).

Experimental results demonstrate the feasibility of the proposed system for greenhouse cluster harvesting and edge-deployable robotic operation, providing an effective solution for intelligent greenhouse tomato harvesting.

## 2 Design of the prototype greenhouse tomato harvesting robot and its hardware system based on YOLOv8n and ROS

### 2.1 Overall functional design

The proposed system is a robot for greenhouse tomatoes that is employed to detect maturity for cluster picking. The end-effector adopts a double-claw structure with a soft claw and pneumatic scissors (Yin et al., 2025).This design balances grasping stability and picking efficiency while reducing mechanical damage to the tomato cluster (Navas et al., 2024).The overall functions of the greenhouse tomato cluster harvesting robot are shown in **Figure 1**, while the overall functional workflow of the proposed system is shown in **Figure 2** depth camera is used to capture greenhouse images and obtain depth information, and the proposed YOLOv8n-BiFPN-WIoU network is adopted to realize tomato-cluster detection and maturity classification. The detected tomato clusters were subsequently localized in three dimensions using aligned RGB-D data, and the resulting target pose was used to guide grasping and cutting. The detailed localization and pose-generation procedure is described in Section 3.4.The manipulator adopts a series of multi-joint structures, providing a range of ±60°, the middle arm extends and retracts by 300 mm, and the wrist rotates by ±180°, thus creating a spherical workspace that can cover tomato clusters at different heights and distances (Rong et al., 2022). Motion planning is subsequently required to generate feasible and collision-aware trajectories for harvesting execution (Shinde et al., 2024).A pressure-sensing and cushioning module was integrated into the fruit-collection system for auxiliary fruit handling.

**Figure 1:**
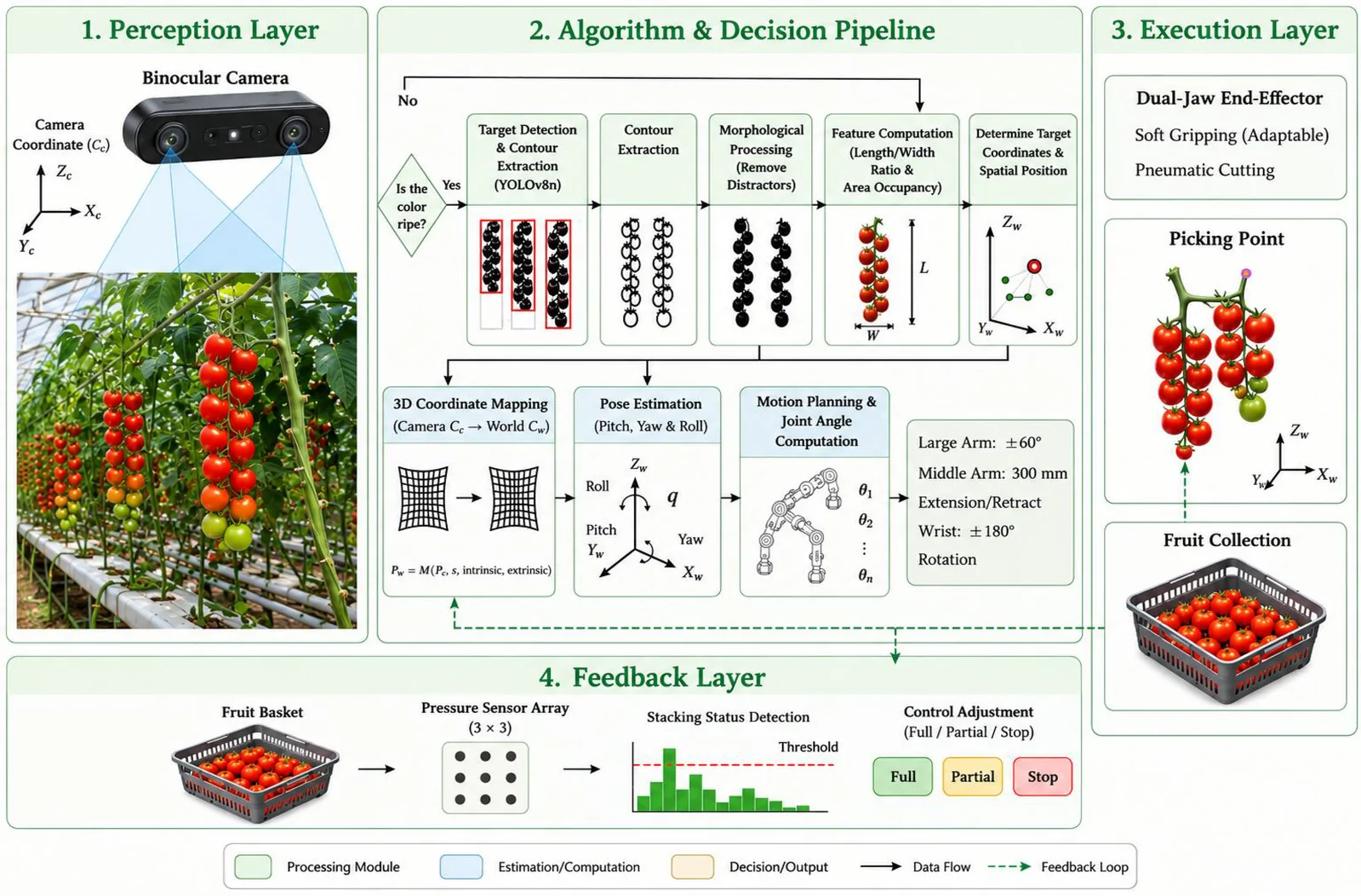
Overall architecture of the intelligent greenhouse tomato cluster harvesting robot system.

**Figure 2:**
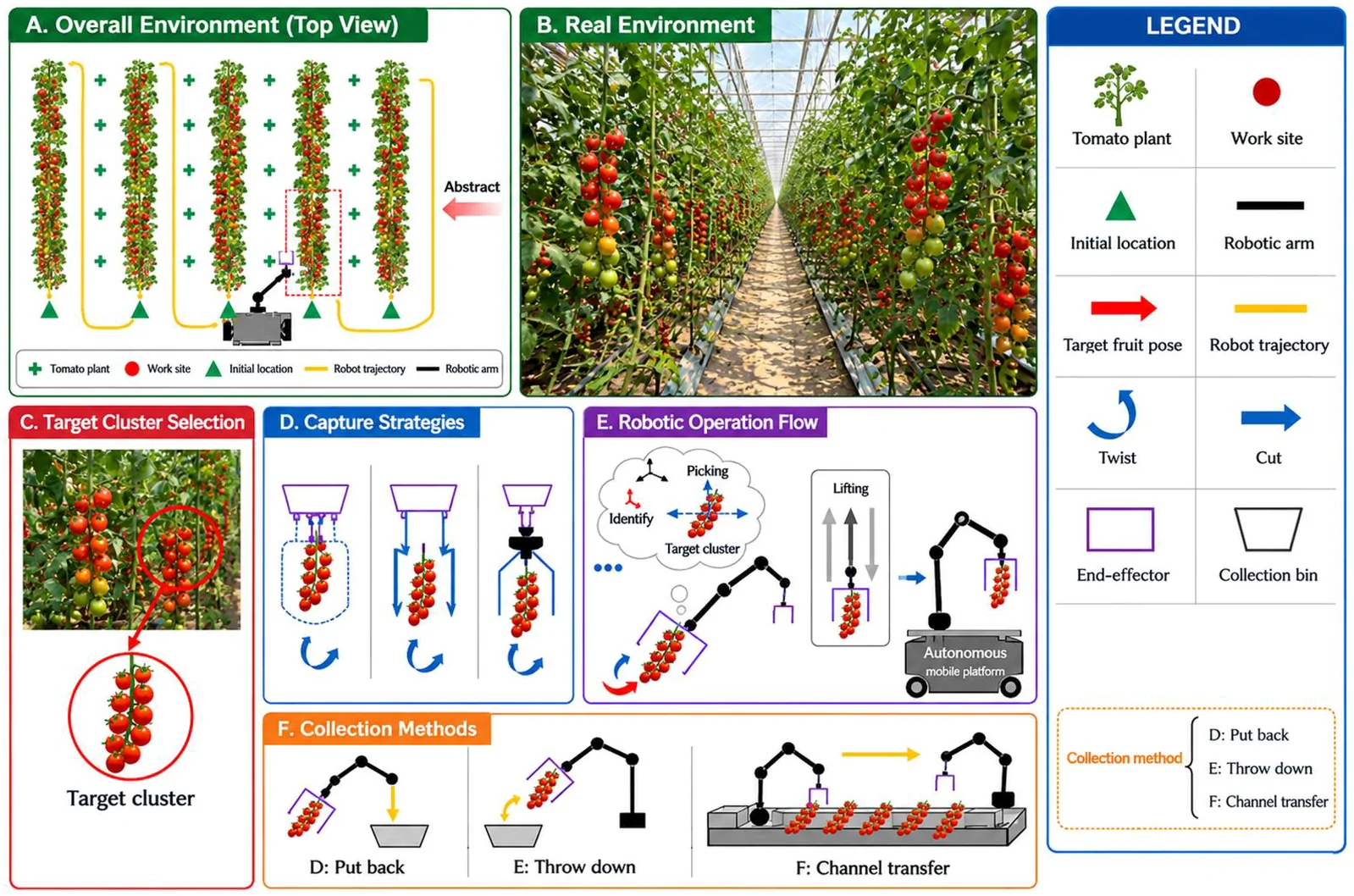
Overall function of the greenhouse tomato-cluster-harvesting robot.

### 2.2 Robot structural design

The greenhouse tomato cluster harvesting robot consists of three main modules: the harvesting execution module, the mobile support module, and the intelligent control module. The main structural components of the harvesting robot are shown in **Figure 3**. The harvesting execution module is responsible for tomato cluster perception, target localization, grasping, cutting, and fruit collection. It mainly includes the Intel RealSense D435 depth camera, six-axis robotic arm, dual-claw end-effector, pneumatic cutting unit, and loading platform. The integrated hardware system of the harvesting robot is presented in **Figure 4**. The D435 depth camera provides depth-based visual information for target positioning, while the robotic arm and end-effector complete the grasping and cutting operations. The mobile support module adopts a rail-guided chassis, which enables the robot to move stably between predefined harvesting stations along fixed greenhouse rails. The rail network provides a predefined and physically constrained motion space for the robot, while the ROS-based control system autonomously manages station-to-station movement according to the harvesting task, encoder feedback, and station-position information. Therefore, the proposed system performs rail-guided station-to-station motion with precise stopping at predefined harvesting stations rather than unconstrained free-space navigation. The intelligent control module is responsible for data processing, command transmission, and multi-module coordination. It receives depth-camera perception information, sensor feedback, and robotic arm status signals, and coordinates the rail-guided chassis, robotic arm, end-effector, and loading platform through the ROS-based control framework. The prototype was powered by a 48 V, 32 Ah LiFePO_4_ battery, providing at least 3 h of operation under normal harvesting conditions.

**Figure 3:**
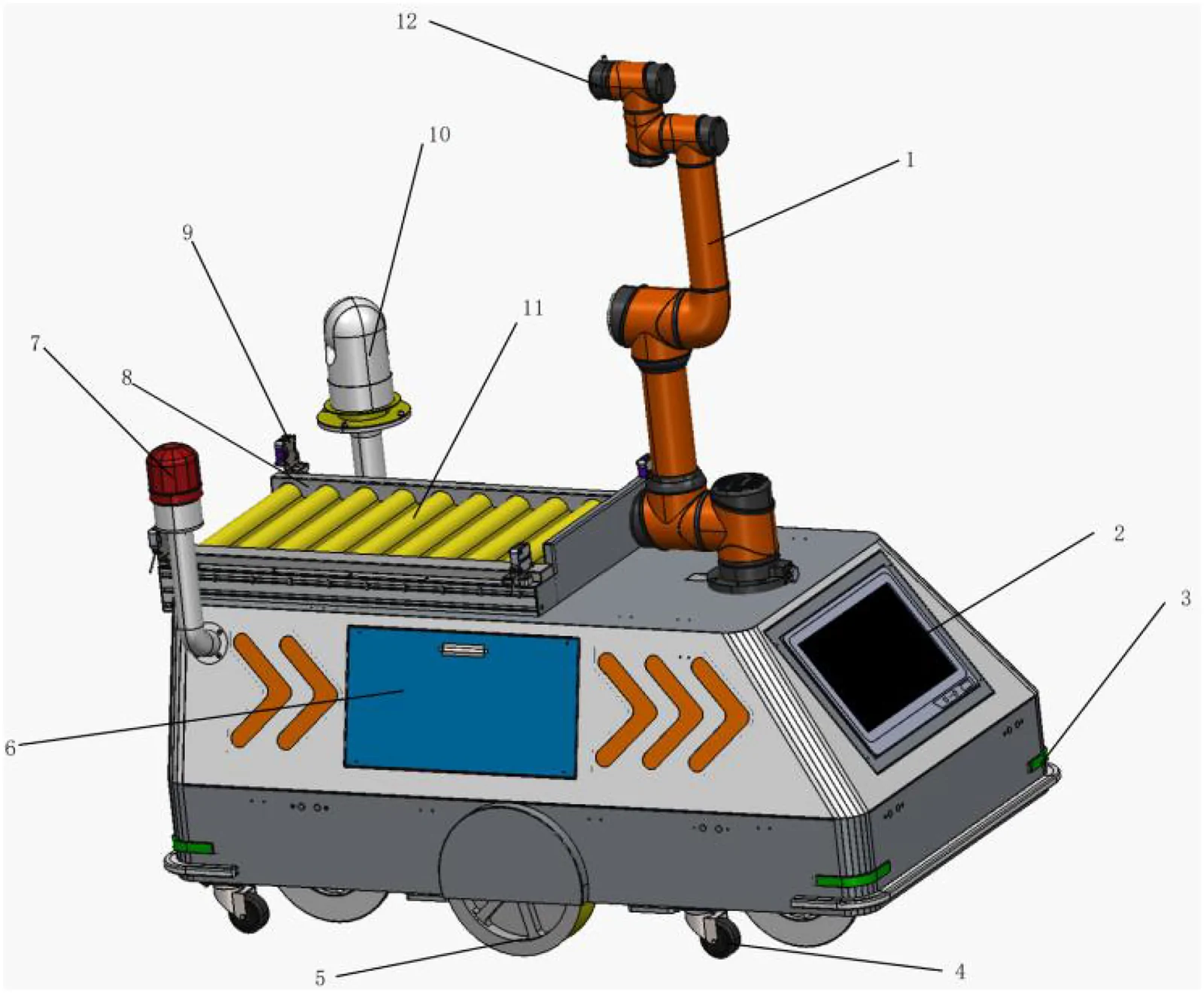
Overall structure diagram. 1.Six-axis robotic arm 2. Touchscreen control panel 3. Protective light strip 4. Guide wheel 5. Rail wheel 6. Electrical cabinet 7. Warning light 8. Fruit collection platform 9. Signal sensor 10. Sensor support column 11. Conveyor roller platform 12. End-effector mounting position.

**Figure 4:**
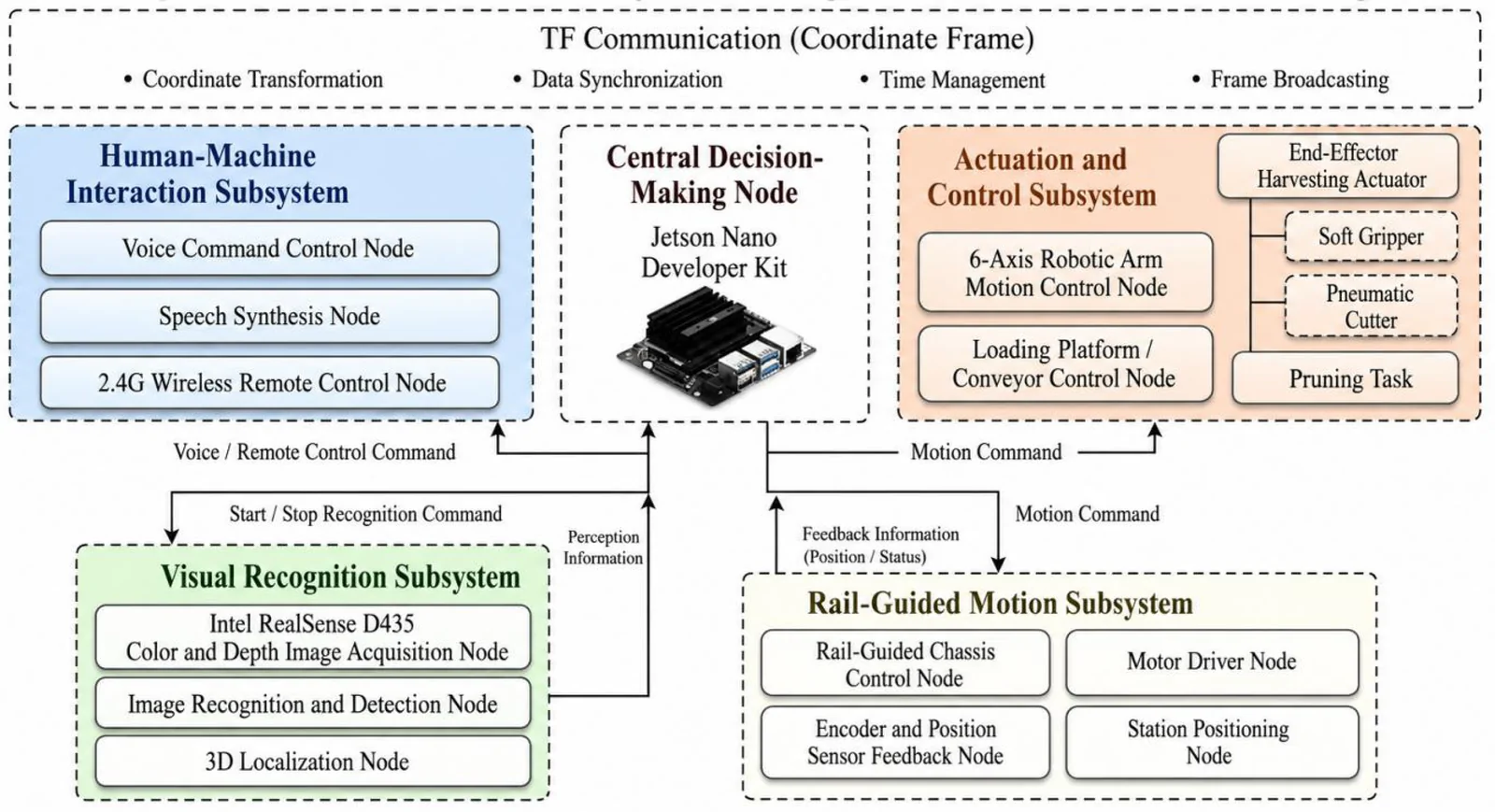
Hardware system of the greenhouse-tomato-cluster-harvesting robot.

### 2.3 Robot control system design

The control system was implemented using a distributed ROS architecture in which perception, target localization, rail-guided motion, manipulation, and safety supervision were organized as independent functional nodes coordinated by a central harvesting state machine (Wang S. et al., 2023). ROS TF2 was used to maintain the spatial relationships among the camera, manipulator, end-effector, and rail-guided base during target localization and execution. The main functional groups were rail-guided station motion, visual perception and localization, harvesting execution, and human–machine interaction, with their principal functions and nominal operating frequencies summarized in **Table 1**.

**Table 1:** Main ROS nodes and nominal operating frequencies.

Functional module	ROS node or core topic	Nominal frequency	Main function
Stereo Camera	/stereo_camera_node	30 Hz	RGB-D image acquisition
Tomato Detection	/tomato_detector	15 Hz	Tomato detection and maturity recognition
3D Pose Estimation	/tomato_pose_estimator	10 Hz	Depth fusion and 3D localization
Target Tracking and Selection	/tomato_target_tracker	10 Hz	Target filtering and selection
Harvest FSM	/harvest_fsm	20 Hz	Harvest-task coordination
Rail-Guided Motion Control	/move_base,/robot_localization	10–50 Hz	Rail motion control and station positioning
MoveIt Motion Planning	/move_group	Event-triggered	Manipulator trajectory planning
Arm Trajectory Control	/arm_controller	100 Hz	Joint trajectory execution
Pneumatic Soft Gripper	/gripper_controller	50 Hz	Gripper control and feedback
Safety Supervision	/safety_supervisor	50 Hz	Fault monitoring and safe stop
TF2 Coordinate Transform	/tf,/tf_static	Dynamic/static	Coordinate-frame transformation

ROS nodes operate asynchronously at different update rates, while TF2 and message timestamps maintain spatial and temporal consistency among perception, decision, and execution modules.

As shown in **Figure 5**, the harvesting robot adopts a distributed ROS software architecture coordinated by the finite-state-machine node/harvest_fsm. The D435 acquisition node/stereo_camera_node publishes RGB-D data at 30 Hz, while/tomato_detector performs tomato detection and maturity recognition at approximately 15 Hz. The detection results are fused with depth data by/tomato_pose_estimator, which updates the target three-dimensional pose at 10 Hz. After temporal validation and target selection, the valid target is transmitted to/harvest_fsm, which operates at 20 Hz and coordinates rail-guided station motion, Moveit trajectory planning, manipulator execution, grasping/cutting, and fruit placement. The manipulator trajectory controller operates at 100 Hz, while end-effector feedback and independent safety supervision are updated at 50 Hz. ROS TF2 provides the transformation relationships among the rail/base, manipulator, end-effector, and D435 camera frames. Before a target pose is used for trajectory planning, both its time stamp and the corresponding TF transformation are checked to prevent stale or spatially inconsistent perception data from being executed.

**Figure 5:**
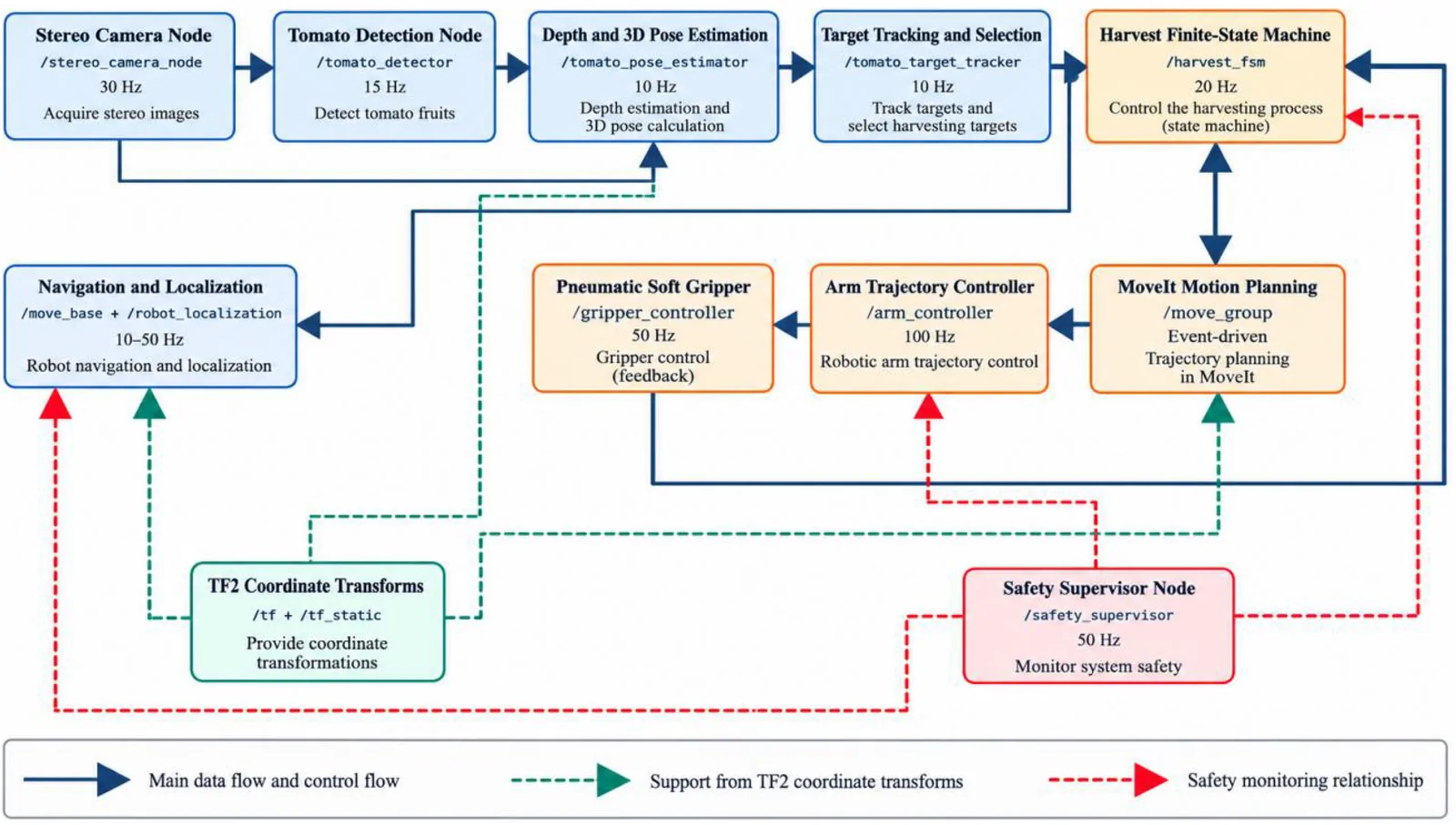
ROS-based software architecture and information flow of the greenhouse tomato harvesting robot.

## 3 Harvesting execution module design

This study adopts a double-claw cluster harvesting method for tomatoes, which combines flexible clamping and pneumatic shearing. The soft claw wraps around the fruit stalk with three-point contact and works with the unidirectional rotary cutting of the pneumatic scissors. Compared with the traditional rigid grasping method, this method requires lower positioning accuracy, and a single robotic arm can complete the entire process of identification, grasping, cutting, and collection. The proposed visual system provides a three-dimensional cluster-level target reference rather than an independently detected fruit-stalk keypoint. After the manipulator reaches the predefined approach pose, the compliant lower claw accommodates the residual localization error and captures the accessible stem region. The pneumatic scissors are mechanically integrated above the lower claw so that grasping and cutting are completed sequentially within the predefined end-effector geometry. This avoids the pulling damage caused by the traditional cluster-harvesting method and reduces the damage rate.

### 3.1 Dual-claw mechanism design

Given that the cluster shape of greenhouse tomatoes is irregular and the stem is slender and easy to damage, a double-claw composite structure combining flexible clamping and pneumatic shearing was adopted (Gao et al., 2022),as shown in **Figure 6**. The lower claw body, made of food-grade silicone material, has a surface with a 0.5 mm anti-slip texture and can be used with tomato stems that are 5–12 mm in diameter. The lower claws are unevenly distributed in the circumferential direction, and flexibly deform to fit the stem, resulting in an increased contact area. The upper claw combines a pneumatic shearing mechanism that has a wedge-shaped blade with a blade angle of 15°, which is made of 420 stainless steel and is driven by a pneumatic controller to a piston push rod. The upper and lower claws were fixed using an aluminum alloy bracket with a spacing of 25 mm. The precision of the internal angle sensor was ±2, which detected the relative angle in real time with respect to the stem, thereby ensuring that the cutting direction formed a 30° angle with the stem. The shearing force required is reduced by using the lever principle, and thus, the pulling damage to the fruits is reduced. The key structural and control parameters of the proposed dual-claw end-effector are summarized in **Table 2**.

**Figure 6:**
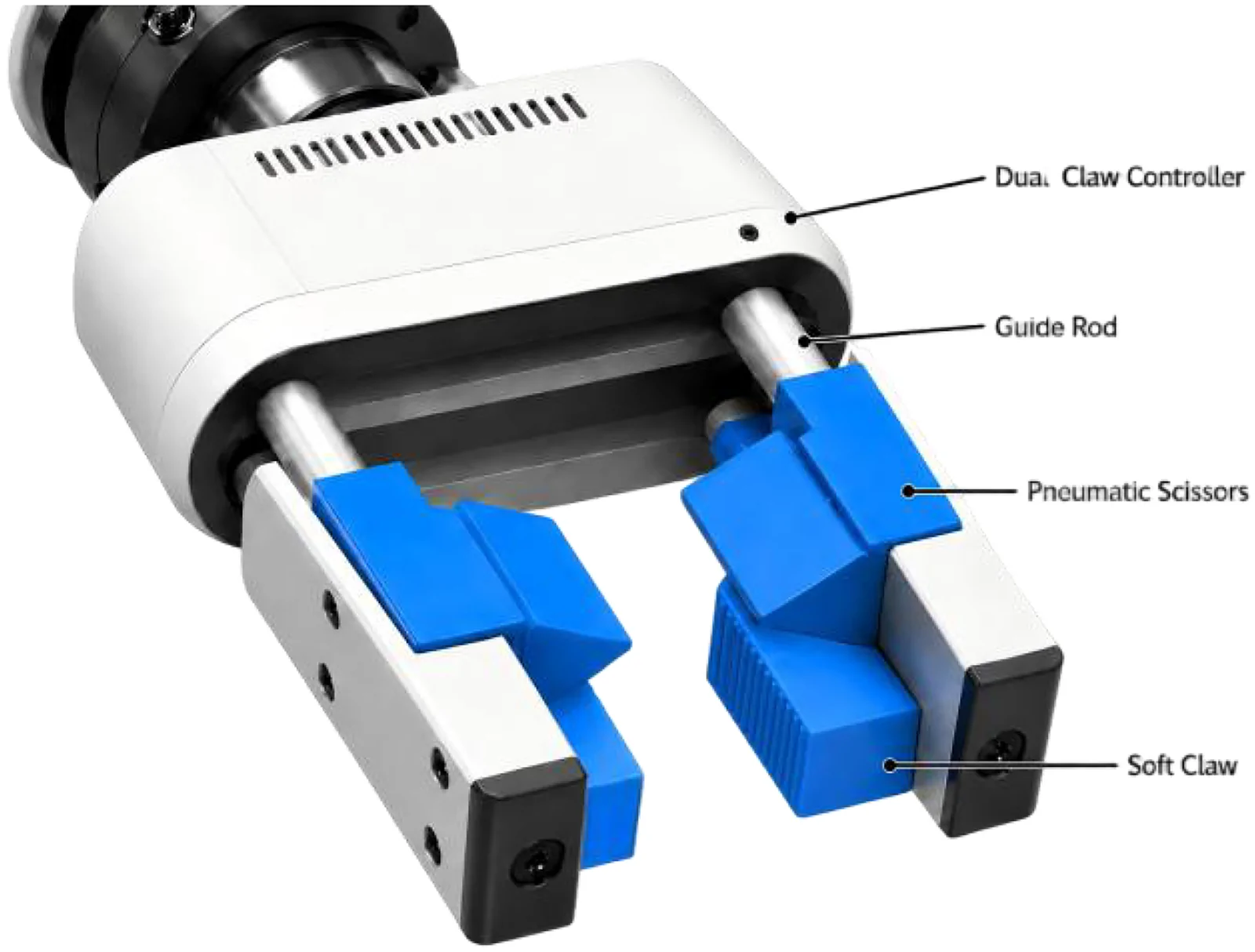
Schematic of the dual-claw structure. 1. Dual-Claw Controller 2. Pneumatic Controller 3. Pneumatic Scissors 4. Soft Claw 5. Drive Motor 6. Gear Shaft.

**Table 2:** Summary of key mechanical and control parameters of the dual-claw system.

Parameter	Value	Unit	Material
Claw spacing	25	mm	Aluminum alloy
Opening angle	35	°	Aluminum alloy
Soft claw hardness	50	Shore A	Silicone rubber
Soft claw elastic modulus	1.2	MPa	Silicone rubber
Surface texture depth	0.5	mm	Silicone rubber
Stem diameter range	5–12	mm	—
Blade angle	15		420 stainless steel
Blade thickness	1.2	mm	420 stainless steel
Actuation response time	0.35	s	—

### 3.2 Dual-claw end-effector design and system-level evaluation

During harvesting, the lower compliant claw first contacted and retained the stem, after which the upper pneumatic scissors completed cutting and fruit transfer. The end-effector was evaluated as part of the integrated robotic system through harvesting success, fruit damage, false cutting, and harvesting cycle time in the greenhouse experiments described in Section 8.

Independent bench tests of clamping force, stem–claw friction, load-retention capacity, and cyclic wear life were not conducted in the present study. Consequently, the reported harvesting results represent the combined effects of visual localization, hand–eye transformation, manipulator positioning, and end-effector execution. The current results therefore cannot quantitatively distinguish failures caused by visual-localization errors from those caused by the intrinsic mechanical limitations of the end-effector.

### 3.3 Six-DOF robotic arm design

#### 3.3.1 Standard D–H modeling and forward kinematics

The six-DOF manipulator was modeled using the standard Denavit–Hartenberg (D–H) convention rather than the modified D–H convention proposed by Craig. The D-H coordinate frames were assigned to the six-DOF manipulator according to the standard D-H convention, as shown in **Figure 7**. For joint i, the ${\text{z}}_{\text{i}-1}-\text{axis}$ was aligned with the corresponding joint axis, and the transformation from frame {i−1} to frame {i} was performed in the order ${\text{R}}_{\text{z}}({\text{θ}}_{\text{i}}){\text{T}}_{\text{z}}({\text{d}}_{\text{i}}){\text{T}}_{\text{x}}({\text{a}}_{\text{i}}){\text{R}}_{\text{x}}({\text{α}}_{\text{i}})$as defined in Equation 1. The parameters ${\text{a}}_{\text{i}},{\text{α}}_{\text{i}},{\text{d}}_{\text{i}},$ and ${\text{θ}}_{\text{i}}$ denote the link length, link twist, link offset, and joint angle, respectively. The standard D–H parameters and joint limits of the manipulator are summarized in **Table 3**.

**Table 3:** Standard D–H parameters of the six-DOF manipulator.

i	α_i_ (°)	a_i_ (mm)	d_i_ (mm)	θ_i_	Variable range
1	90	0	90	θ_1_	-360° ~ 360°
2	0	425	0	θ_2_	-90° ~ 90°
3	0	390	0	θ_3_	-180° ~ 180°
4	90	0	110	θ_4_	-30° ~ 30°
5	-90	0	90	θ_5_	-90° ~ 90°
6	0	0	80	θ_6_	-90° ~ 90°

The standard D–H convention, rather than the modified D–H convention proposed by Craig, was adopted throughout this study. Accordingly, the transformation from frame {i−1} to frame {i} follows the order ${\text{R}}_{\text{z}}({\text{θ}}_{\text{i}}){\text{T}}_{\text{z}}({\text{d}}_{\text{i}}){\text{T}}_{\text{x}}({\text{a}}_{\text{i}}){\text{R}}_{\text{x}}({\text{α}}_{\text{i}})$.

**Figure 7:**
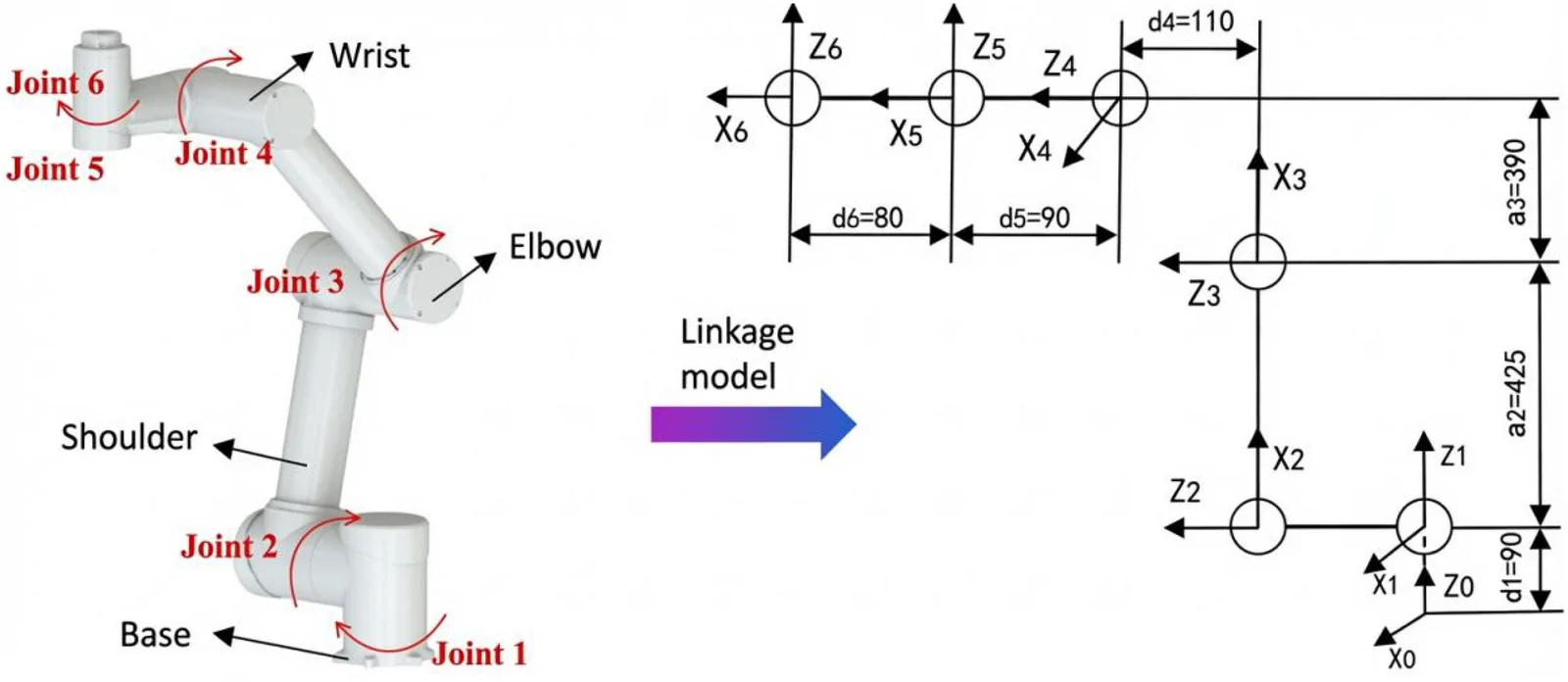
Coordinate-frame assignment of the six-DOF manipulator based on the standard D–H convention.

(1) $${i-1}_{T}^{i}=R_{z}(\theta_{i})T_{z}(d_{i})T_{x}(a_{i})R_{x}(\alpha_{i})$$

Substituting the parameters in **Table 3** into the standard D–H formulation yielded the joint transformation matrices and the overall base-to-end-effector transformation. Where the tomato harvesting robot equation of motion $6_{0}^{\text{T}}$ is expressed as the position and orientation of the end-effector with respect to the base coordinate system:

(2) $$0_{T}^{6}=0_{T}^{1}1_{T}^{2}2_{T}^{3}3_{T}^{4}4_{T}^{5}5_{T}^{6}=[\begin{array}{cc} R_{3\times 3} & P\\ 0 & 1 \end{array}]$$

Here, R denotes the end-effector orientation and $\text{p}={\left[\begin{array}{ccc} {\text{p}}_{\text{x}} & {\text{p}}_{\text{y}} & {\text{p}}_{\text{z}} \end{array}\right]}^{\text{T}}$ denotes its position in the robot base frame. The transformation in (Equation 2) was used both for forward-position calculation and for verification of the inverse-kinematic solutions.

#### 3.3.2 Inverse kinematics and model validation

For a desired end-effector pose $0_{\text{T}}^{6,\text{d}}$, the inverse-kinematic problem is expressed by Equations 3 and 4.

(3) $$0_{T}^{6}(\theta ){=}_{0}^{T6,d}$$

(4) $$\theta ={\left[\theta_{1},\theta_{2},\theta_{3},\theta_{4},\theta_{5},\theta_{6}\right]}^{T}.$$

The inverse-kinematic calculation was performed using the same D–H model defined in **Table 3**. Inverse-kinematic solutions were filtered according to the joint limits in **Table 3** and verified using the forward-kinematic model in (Equation 2).

When multiple feasible solutions were available, the solution with the smallest joint displacement from the current configuration was selected for MoveIt-based trajectory planning and execution.

Five representative target positions within the harvesting workspace were used to validate the D–H model. For each target, the calculated end-effector position was compared with the measured position after execution.

The positioning errors along the three Cartesian axes were defined according to Equation 5, and the three-dimensional positioning error was calculated according to Equation 6.

(5) $$e_{x}=|x_{m}-x_{c}|,e_{y}=|y_{m}-y_{c}|,e_{z}=|z_{m}-z_{c}|,$$

and the three-dimensional positioning error was calculated as

(6) $$e_{p}=\sqrt{e_{x}^{2}+e_{y}^{2}+e_{z}^{2}},$$

Where $\left({\text{x}}_{\text{c}},{\text{y}}_{\text{c}},{\text{z}}_{\text{c}}\right)$ denotes the calculated end-effector position and $\left({\text{x}}_{\text{m}},{\text{y}}_{\text{m}},{\text{z}}_{\text{m}}\right)$ denotes the corresponding measured position.

**Table 4:** Validation of the six-DOF manipulator kinematic model.

Target	Calculated position (mm)	Measured position (mm)	Error (mm)
P1	(350, 100, 450)	(352.1, 98.7, 452.4)	3.45
P2	(420, −100, 400)	(417.8, −101.5, 402.1)	3.39
P3	(500, 50, 500)	(502.6, 51.2, 497.9)	3.55
P4	(300, −150, 550)	(297.6, −148.1, 552.8)	4.14
P5	(520, 120, 350)	(522.2, 117.8, 352.5)	3.99
Mean ± SD	—	—	3.70 ± 0.32

The calculated and measured positions showed good agreement across the five representative targets, with a three-dimensional positioning error of 3.39–4.14 mm and a mean error of 3.70 ± 0.32 mm. These results indicate that the D–H model provides an adequate geometric basis for subsequent MoveIt trajectory planning and harvesting execution.

#### 3.3.3 Manipulator trajectory planning and simulation analysis

Based on the established kinematic model, joint-space motion was analyzed using quintic polynomial interpolation in MATLAB. MoveIt was used for collision checking and executable trajectory generation in the ROS-based system, whereas the MATLAB simulation was used only to evaluate trajectory continuity and smoothness. After the rail-guided chassis reached the harvesting station, the target pose was transformed into the robot base frame for manipulator planning.

The reachable workspace of the six-DOF manipulator was calculated from the kinematic model. **Figure 8** shows the three-dimensional workspace and its X–Z, Y–Z, and X–Y projections. The calculated workspace covered the representative region used in the subsequent harvesting simulation.

**Figure 8:**
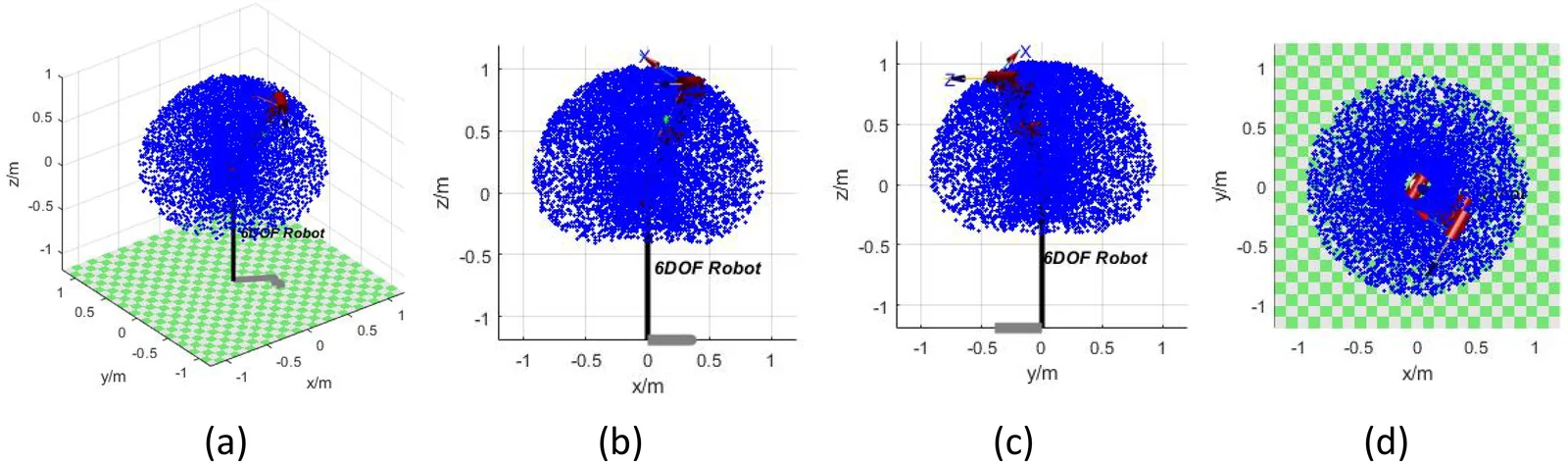
Reachable workspace of the six-DOF robotic arm. **(A)** three-dimensional view **(B)** X–Z plane projection **(C)** Y–Z plane projection **(D)** X–Y plane projection.

For each joint, a quintic polynomial was used to generate continuous position, velocity, and acceleration profiles, as expressed in Equation 7.

(7) $$\{\begin{array}{c} \theta_{i}(t)=a_{i0}+a_{i1}t+a_{i2}t^{2}+a_{i3}t^{3}+a_{i4}t^{4}+a_{i5}t^{5}\\ {\dot{\theta }}_{i}(t)=a_{i1}+2a_{i2}t+3a_{i3}t^{2}+4a_{i4}t^{3}+5a_{i5}t^{4}\\ {\ddot{\theta }}_{i}(t)=2a_{i2}+6a_{i3}t+12a_{i4}t^{2}+20a_{i5}t^{3} \end{array}$$

where $\theta_{i}(t)$, ${\dot{\theta }}_{i}(t)$, and ${\ddot{\theta }}_{i}(t)$ denote the angular displacement, angular velocity, and angular acceleration of the i-th joint, respectively, and $a_{i0}$– $a_{i5}$ are the corresponding polynomial coefficients.

To satisfy the smooth start and stop requirements of the harvesting motion, six boundary conditions were set for each joint, including the initial angle, final angle, initial velocity, final velocity, initial acceleration, and final acceleration. The boundary conditions are expressed in Equation 8.

(8) $$\{{\dot{\begin{array}{c} \theta_{i}(0)=\theta_{i0},\;\theta_{i}(tf)=\theta_{if}\\ {\dot{\theta }}_{i}(0)={\dot{\theta }}_{i0},\;{\dot{\theta }}_{i}(tf)={\dot{\theta }}_{if}\\ {\ddot{\theta }}_{i}(0)={\ddot{\theta }}_{i0},\;{\ddot{\theta }}_{i}(tf)={\ddot{\theta }}_{if} \end{array}}}_{i}$$

The boundary conditions included the initial and final joint positions, velocities, and accelerations.For the offline trajectory analysis, the initial and terminal joint velocities and accelerations were set to zero to ensure smooth start and stop conditions.

Normalized time τ=t/T, 0≤τ≤1, was used for offline trajectory analysis. The simulation evaluated trajectory continuity rather than reproducing the physical execution time; the actual execution time was determined by MoveIt according to the planned path and manipulator constraints. The corresponding experimental results are summarized in **Table 4**.

A representative feasible initial and target configuration was selected for trajectory simulation. The corresponding joint boundary conditions are listed in **Table 5**, with zero initial and terminal velocities and accelerations.

**Table 5:** Representative boundary conditions for six-DOF joint trajectory simulation.

Joint	Initial angle (°)	Final angle (°)	Initial velocity (° s^-^¹)	Final velocity (° s^-^¹)	Initial acceleration (° s^-^²)	Final acceleration (° s^-^²)
Joint1	0	28	0	0	0	0
Joint2	-15	-42	0	0	0	0
Joint3	35	58	0	0	0	0
Joint4	0	18	0	0	0	0
Joint5	20	35	0	0	0	0
Joint6	0	-25	0	0	0	0

MATLAB was used to generate the joint position, velocity, and acceleration profiles under the boundary conditions in **Table 5**.

As shown in **Figure 9**, all six joints exhibited continuous displacement, velocity, and acceleration profiles, with zero initial and terminal velocities and accelerations. These results confirm that the selected quintic interpolation satisfied the prescribed boundary conditions and produced smooth point-to-point motion.

**Figure 9:**
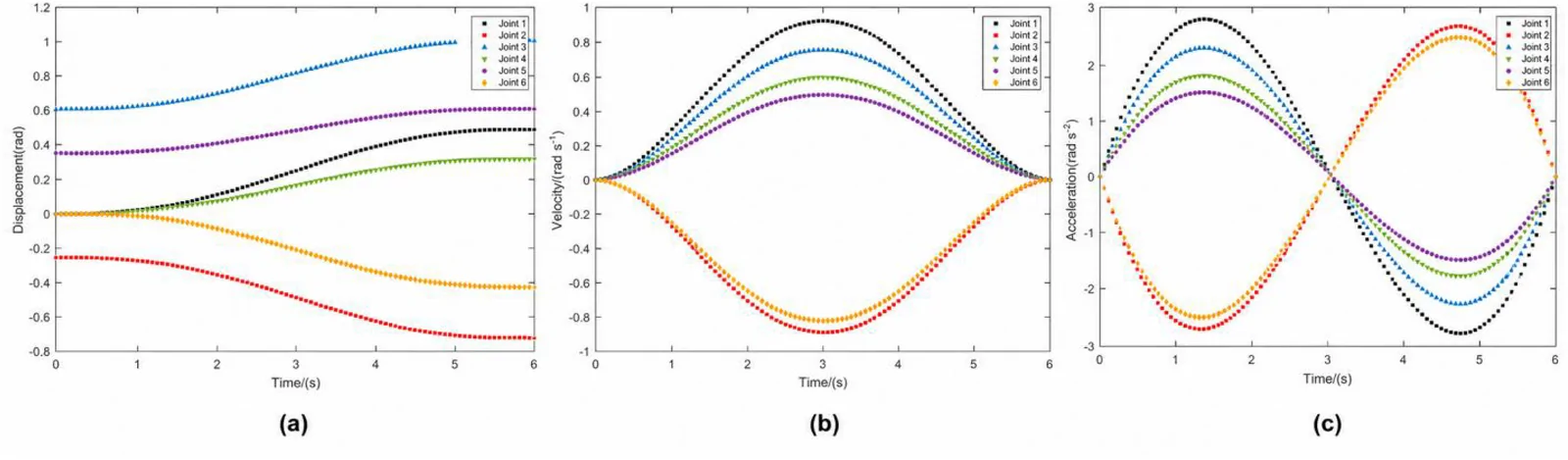
Quintic-polynomial joint trajectories of the six-DOF manipulator. **(a)** angular displacement **(b)** angular velocity **(c)** angular acceleration.

A simplified MATLAB harvesting scene was constructed to further examine the spatial feasibility of the representative motion. Four characteristic configurations from the initial posture to the harvesting posture are shown in **Figure 10**.

**Figure 10:**
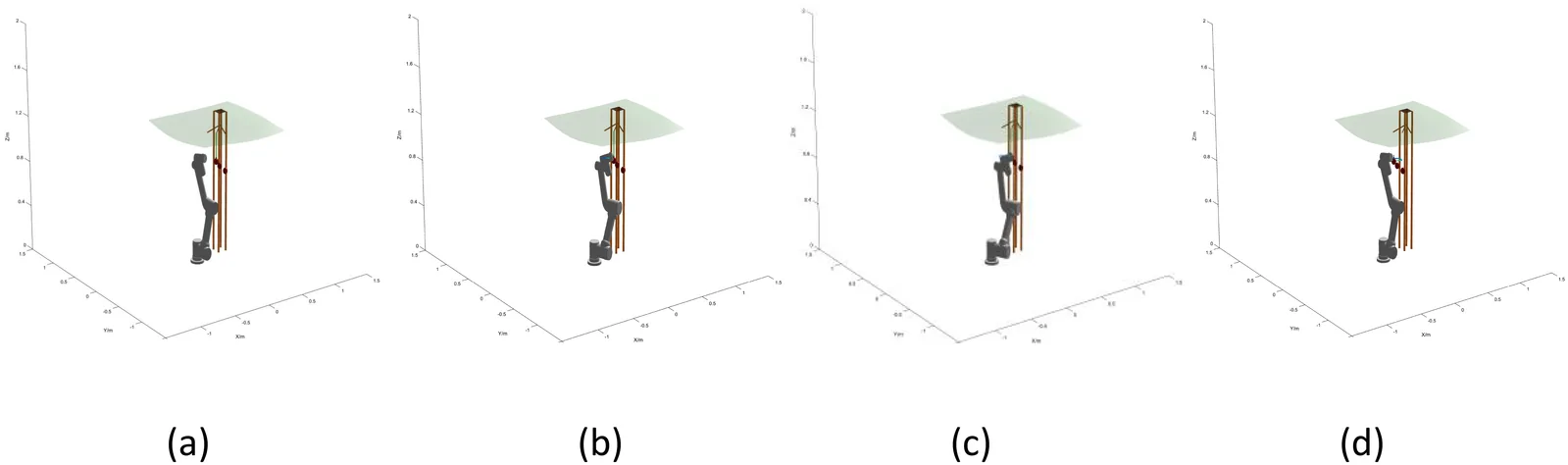
Simulated motion sequence of the six-DOF manipulator during tomato-cluster harvesting. **(a)** initial configuration **(b)** approach toward the target cluster **(c)** end-effector alignment with the harvesting region **(d)** harvesting configuration.

The simulated motion remained within the calculated workspace and maintained continuous joint trajectories throughout the representative harvesting sequence. The offline simulation therefore provided a supplementary check of kinematic feasibility before physical MoveIt execution.

### 3.4 RGB-D calibration and three-dimensional localization

An Intel RealSense D435 RGB-D camera was used for three-dimensional localization of tomato clusters. The camera adopts active stereo depth sensing and was configured at a depth resolution of 1280 × 720 pixels. The nominal depth field of view is approximately 85.2° × 58°, and the effective operating range is approximately 0.3–3.0 m. Before the experiments, the RGB and depth streams were spatially aligned using the Intel RealSense SDK. The intrinsic parameters of the active camera profile are summarized in **Table 6**. The intrinsic parameters provided by the active camera profile were used for three-dimensional reconstruction. The present study did not retain the numerical intrinsic-parameter record as an independent experimental output. These parameters were subsequently used to reconstruct the three-dimensional coordinates of the detected tomato-cluster reference points in the camera coordinate frame. For a target pixel (u,v)with aligned depth $Z_{c}$, its coordinates in the camera coordinate system were calculated according to Equations 9–11.

**Table 6:** D435 configuration and calibration settings.

Parameter	Value
Camera	Intel RealSense D435
Depth technology	Active stereo
Depth resolution	1280 × 720
Depth FOV	85.2° × 58°
Operating range	0.3–3.0 m
Calibration target	ChArUco board
Hand–eye configuration	Eye-in-hand
Calibration poses	20
Test distances	0.4/0.6/0.8 m
Measurements per condition	30

(9) $$X_{c}=\frac{(u-c_{x})Z_{c}}{f_{x}}$$

(10) $$Y_{c}=\frac{(v-c_{y})Z_{c}}{f_{y}}$$

(11) $$Z_{c}=D(u,v)$$

where D(u,v) denotes the depth measurement corresponding to the target pixel.

An eye-in-hand configuration was used to calibrate the rigid transformation between the D435 camera and the robot end-effector. A ChArUco board was observed at 20 robot poses with different translations and orientations, and the hand–eye calibration was formulated as the AX=XB problem. The resulting transformation matrix was incorporated into the ROS TF tree. The original calibration procedure did not retain the per-pose ChArUco corner observations or the AX=XB transformation residual logs. Therefore, ChArUco reprojection RMSE and AX=XB residual errors cannot be reliably reconstructed from the available experimental records and are not reported here as independent hand–eye calibration metrics. Instead, the practical accuracy of the complete RGB-D-to-robot coordinate transformation chain was evaluated separately using physical target localization experiments, as described below.

The target coordinate in the camera frame was transformed into the robot base frame according to Equation 12.

(12) $$B^{p}=B_{T}^{E}E_{T}^{C}C^{p}$$

where ${\text{E}}_{T}^{\text{C}}$ is obtained from hand–eye calibration and ${\text{B}}_{T}^{\text{E}}$ is calculated from the forward kinematics of the six-DOF manipulator.

The transformed three-dimensional cluster position was defined as the cluster reference point ${\text{P}}_{r}^{\text{b}}$ in the robot base coordinate system. This point was used as a spatial reference rather than directly as the physical grasping or cutting point. The grasping point ${\text{P}}_{\text{g}}$was generated by applying a calibrated geometric offset Δ ${\text{P}}_{\text{g}}$ between the cluster reference point and the dual-claw end-effector, as defined in Equation 13.

(13) $$P_{g}=P_{r}^{b}+\Delta P_{g}$$

The cutting point ${\text{P}}_{\text{s}}$​ was subsequently determined from the grasping point according to the fixed geometric relationship between the compliant lower claw and the pneumatic cutting blade, as defined in Equation 14.

(14) $$P_{s}=P_{g}+\Delta P_{gs}$$

Where Δ ${\text{P}}_{\text{g}}$ ​ denotes the calibrated offset from the cluster reference point to the grasping position, and ${\text{ΔP}}_{\text{gs}}$ denotes the fixed geometric offset between the grasping position and the cutting position. The resulting position and predefined end-effector orientation constituted the target harvesting pose, which was then provided to the inverse-kinematic solver and MoveIt for trajectory planning.

Depth measurement error and the final positioning errors in the robot coordinate system were evaluated separately. For each condition, the absolute errors in the X, Y, and Z directions, together with the three-dimensional Euclidean error, were calculated, and the mean, standard deviation, and maximum error were reported.

Under unobstructed conditions, the mean three-dimensional localization error increased from 7.4 mm at 0.4 m to 11.3 mm at 0.8 m. Fruit-overlap occlusion produced the largest localization error, with a mean 3D error of 16.3 mm at 0.8 m. Among the three coordinate components, the Z-direction error was generally larger than the X- and Y-direction errors, indicating that depth uncertainty was the dominant source of localization error. Nevertheless, the mean 3D localization error remained below 17 mm under all evaluated conditions, supporting the feasibility of the D435-based localization system for greenhouse harvesting.

The resulting target pose, consisting of the transformed three-dimensional cluster position and the predefined end-effector orientation, is then provided to the inverse-kinematic solver and MoveIt motion-planning module for harvesting execution. In this way, the proposed RGB-D localization pipeline establishes a complete coordinate transformation chain from image-space tomato-cluster detection to robot-executable harvesting pose generation.

## 4 Mobile support module design based on ROS

The mobile support module adopts a fixed-rail architecture for autonomous station-to-station harvesting. Unlike free-moving agricultural robots that require unrestricted navigation and continuous path planning, the proposed chassis operates within the physically constrained rail network of the greenhouse. Therefore, the primary control task is not global navigation but accurate station selection, motion initiation, travel-state monitoring, station-arrival detection, and automatic stopping.

The rail-guided chassis integrates the travelling mechanism and manipulator support structure required for stable station-to-station harvesting in the greenhouse. The body frame supports the weight of the robot arm, vision system, control system, and harvested fruit collection platform. The rail wheels and guide wheels are matched with the track to constrain the motion direction and ensure smooth travelling along the greenhouse row. The overall structure of the rail-guided harvesting robot base is shown in **Figure 11**.

**Figure 11:**
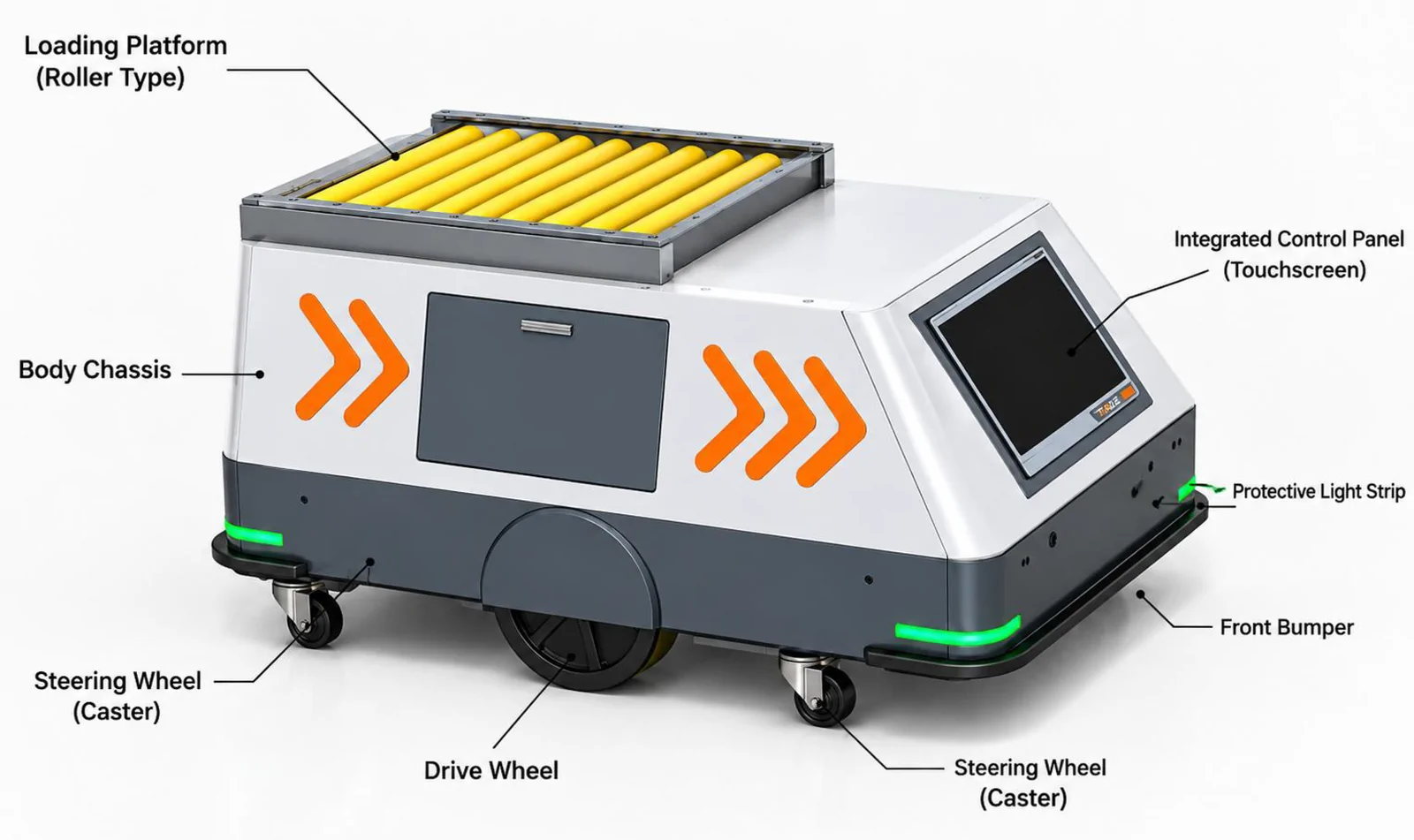
Schematic of the chassis structure.

The rail-guided chassis is driven by a motorized travelling mechanism. The upper computer sends motion commands through the Robot Operating System (ROS), and the lower-level chassis controller controls the motor driver to realize forward movement, stopping, and station positioning. During operation, encoder feedback is used to monitor the travelling speed and displacement of the chassis, while limit switches or position sensors are used to assist in accurate stopping at the target harvesting station. This closed-loop control method improves the repeatability and positioning accuracy of the robot during greenhouse harvesting (Arpitha Shankar and Shivakumar, 2024).

**Figure 12** summarizes the station-to-station motion sequence, including station selection, rail movement, feedback-based arrival detection, and automatic stopping before harvesting execution.

**Figure 12:**
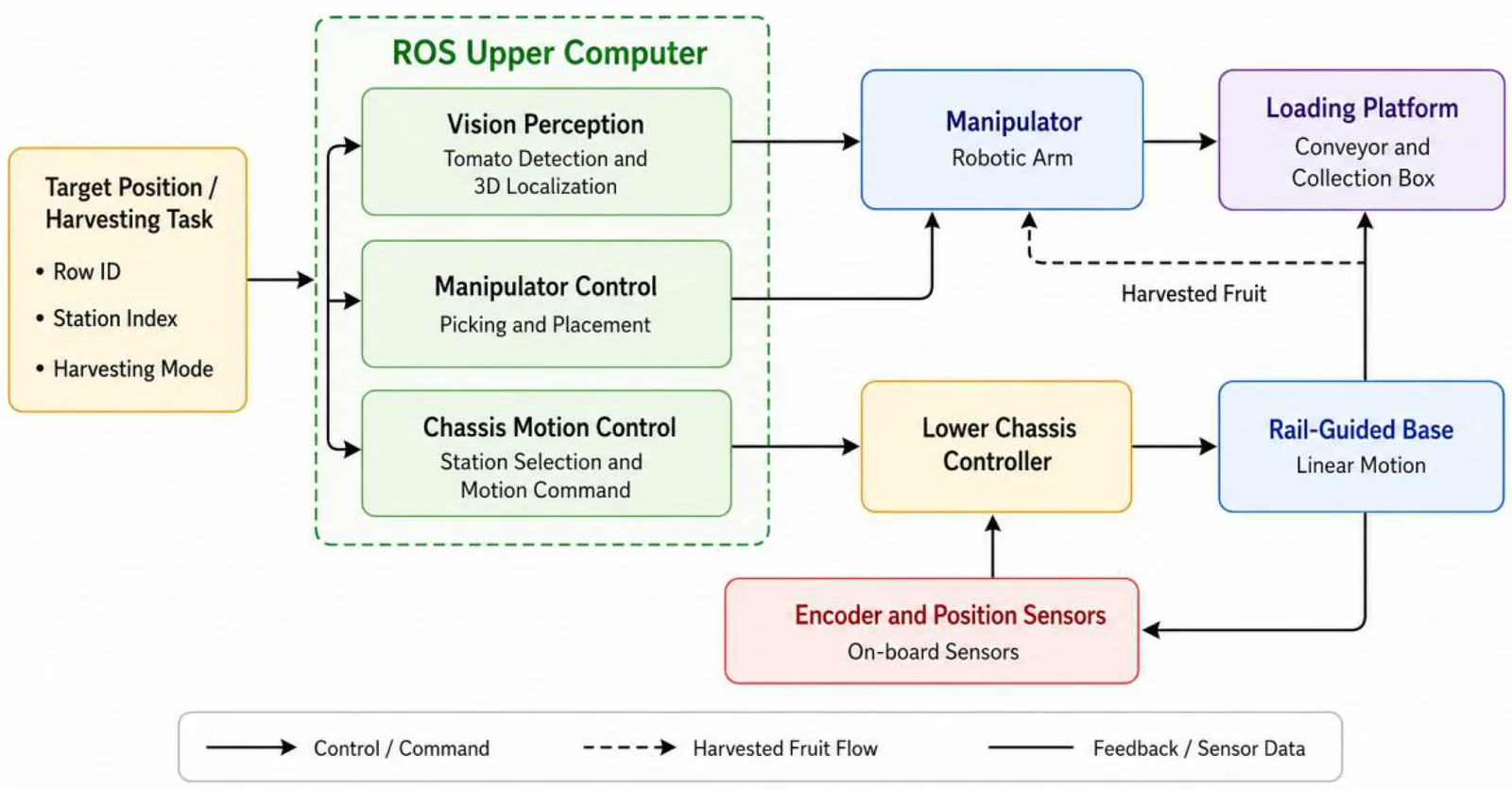
Motion control process of the rail-guided chassis.

The rail-guided base is controlled by the/rail_chassis_controller node. The harvesting state machine issues station-motion commands only when manipulator execution is inactive. Encoder displacement and station-position sensor information are returned to the controller for closed-loop stopping. A station-arrival flag is subsequently transmitted to/harvest_fsm. Visual localization and manipulator planning are enabled only after the chassis has reached the predefined harvesting station and remained stationary.

After reaching a predefined harvesting station, the chassis remained stationary while the RGB-D perception and manipulator modules performed target detection, localization, and harvesting. After task completion, the robot proceeded to the next predefined station.

The rail-guided configuration constrains the mobile motion space and therefore simplifies the base-level control problem while providing repeatable station-to-station positioning.

To verify the motion characteristics of the rail-guided chassis under greenhouse operating conditions, travelling tests were conducted along the fixed rail between predefined harvesting stations. The chassis was commanded by the ROS upper-level controller, while encoder and position-sensor feedback were used for motion-state monitoring and station positioning. As shown in **Figure 13**.

**Figure 13:**
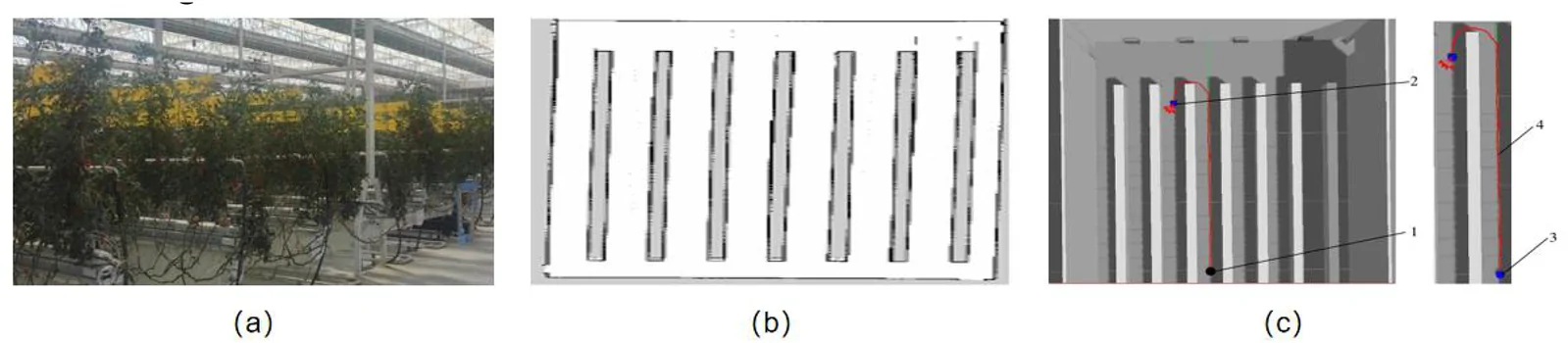
Greenhouse environment and rail-guided travelling verification. **(a)** real scene of tomato cultivation rows in the greenhouse **(b)** greenhouse rail layout **(c)** recorded station-to-station travelling trajectory 1: start point; 2: end point; 3: robot; 4: robot travelling trajectory.

The control framework of the rail-guided greenhouse tomato harvesting robot is shown in **Figure 14**. The ROS upper computer is responsible for task scheduling, vision perception, manipulator control, and chassis motion coordination. The lower-level chassis controller receives velocity or position commands from ROS and controls the rail-guided base to move between harvesting stations.

**Figure 14:**
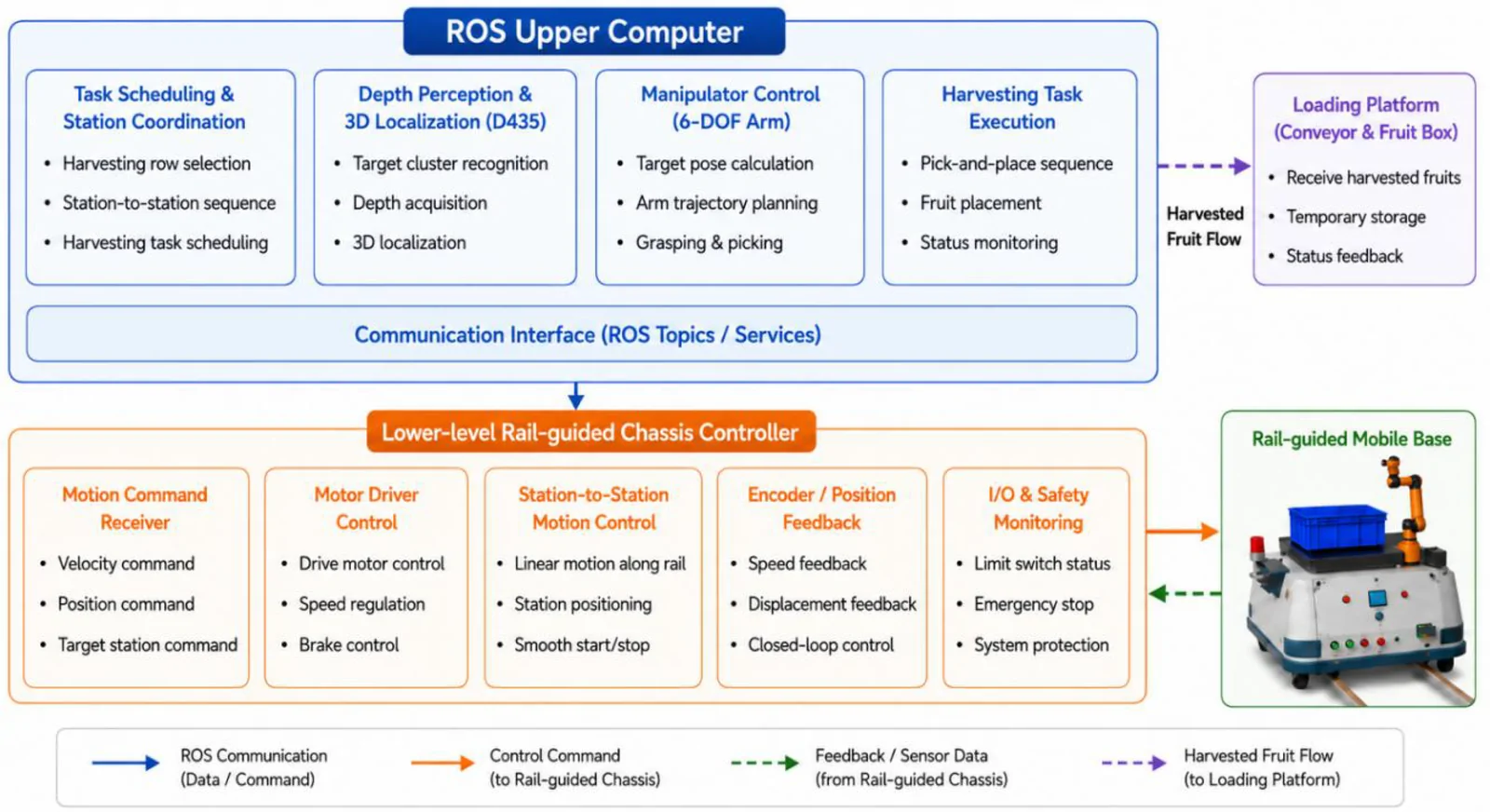
ROS-based control framework for rail-guided chassis motion and harvesting-task coordination.

## 5 Intelligent control module design

The overall harvesting workflow is shown in **Figure 15**. After station arrival, the system performs perception, target validation, pose generation, motion planning, grasping/cutting, fruit placement, and return. Voice or remote commands can also be used to designate harvesting tasks (Singh et al., 2024).

**Figure 15:**
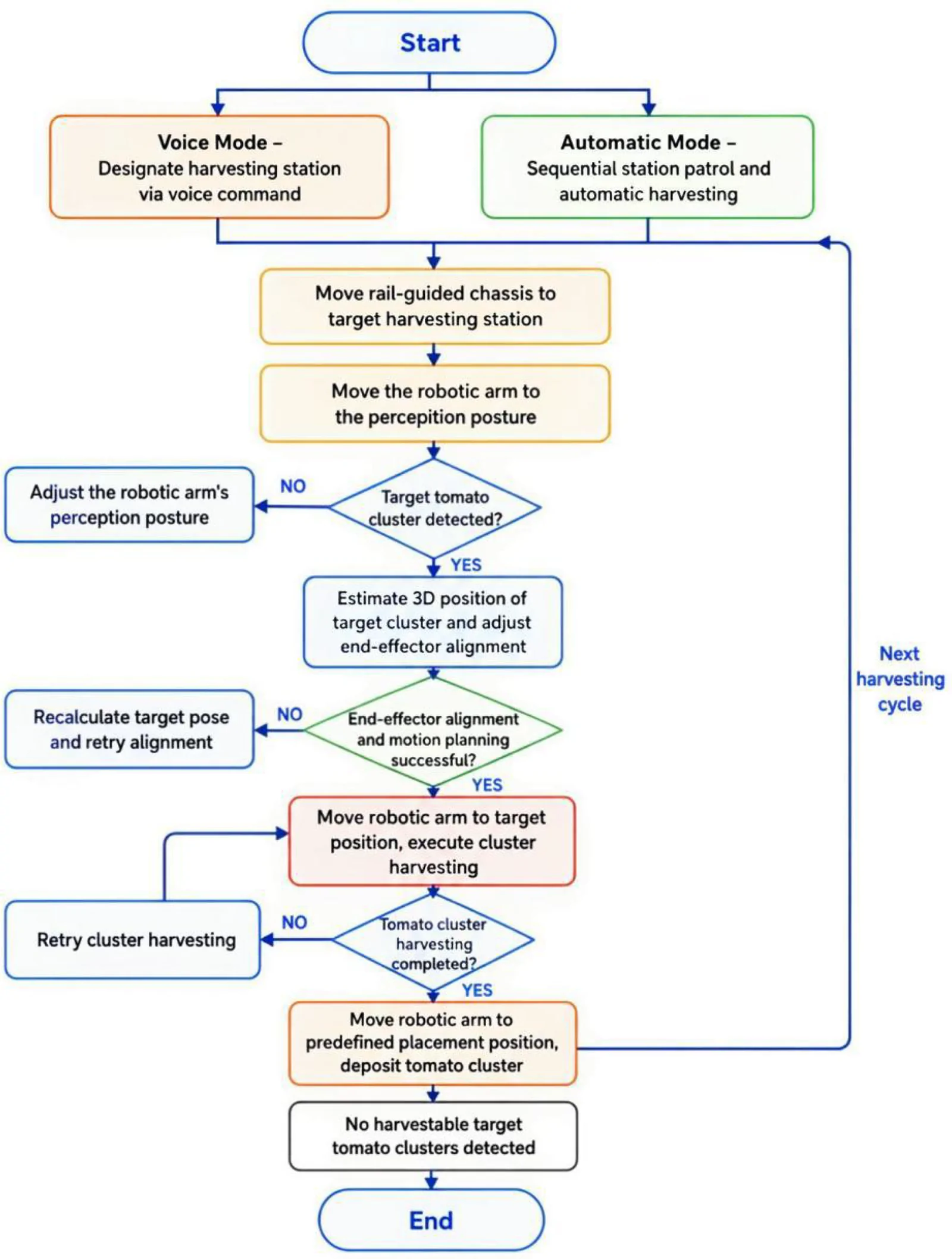
Task flow of the greenhouse tomato-harvesting robot.

Harvest-task coordination was implemented using a finite-state machine managed by/harvest_fsm. The principal state sequence was:idle → move to station → wait stable → perception → target validation → motion planning → approach → grasp/cut → place → return → next station

State transitions were triggered by valid perception, rail-arrival feedback, motion-planning results, manipulator execution status, and end-effector feedback. Target execution was permitted only when the detection result and corresponding TF transformation were valid and sufficiently recent. For a partially occluded cluster, harvesting execution was initiated only when the detection confidence, aligned depth data, and coordinate transformation satisfied the validity checks. If leaf occlusion resulted in a low-confidence detection or invalid depth measurement, the target pose was rejected by the harvesting state machine. The manipulator then adjusted the perception posture and reacquired the RGB-D data before repeating target detection and localization. If a reliable target pose could still not be obtained, the cluster was skipped rather than forcing an unsafe harvesting operation.

Fault handling was implemented through/harvest_fsm and the independent/safety_supervisor. Invalid target poses, unavailable TF transformations, planning failures, and execution timeouts triggered controlled rejection or retry, whereas emergency-stop activation, persistent communication loss, excessive joint error, or collision risk caused immediate task cancellation and safe stop.

The/safety_supervisor independently monitored emergency-stop status, communication health, node status, joint-tracking error, actuator state, and pneumatic feedback at 50 Hz. Recoverable faults triggered controlled retry, whereas persistent or safety-critical faults caused immediate chassis stop and manipulator cancellation.

## 6 Maturity detection function design based on YOLOv8n-BiFPN-WIoU

### 6.1 Dataset source and partitioning

The image dataset used in this study was obtained from the publicly available dataset entitled “A dataset of greenhouse cluster tomato fruit in the Tomato Town of Jinzhong National Agricultural High-tech Industries Demonstration Zone in 2022” (Song et al., 2023). The dataset is publicly accessible through the Science Data Bank at https://doi.org/10.57760/sciencedb.05228. It was collected in a glass greenhouse at the Shanxi Nonggu Tomato Town, Gezitou Village, Fancun Town, Taigu District, Jinzhong, Shanxi Province, China. The samples were cluster-type tomatoes of the “Weina” cultivar. Images were captured under sunny and cloudy conditions at different times of day, including morning, noon, afternoon, and dusk. Multiple smartphone models were used to acquire images from different viewpoints and illumination directions, including front-light, back-light, and top-light condition, as shown in **Figure 16**. The dataset contains 3,665 valid original images with a total data volume of approximately 5.31 GB. All dataset statistics reported in this study refer to the original images before data augmentation. Representative images are shown in **Figure 17**.

**Figure 16:**
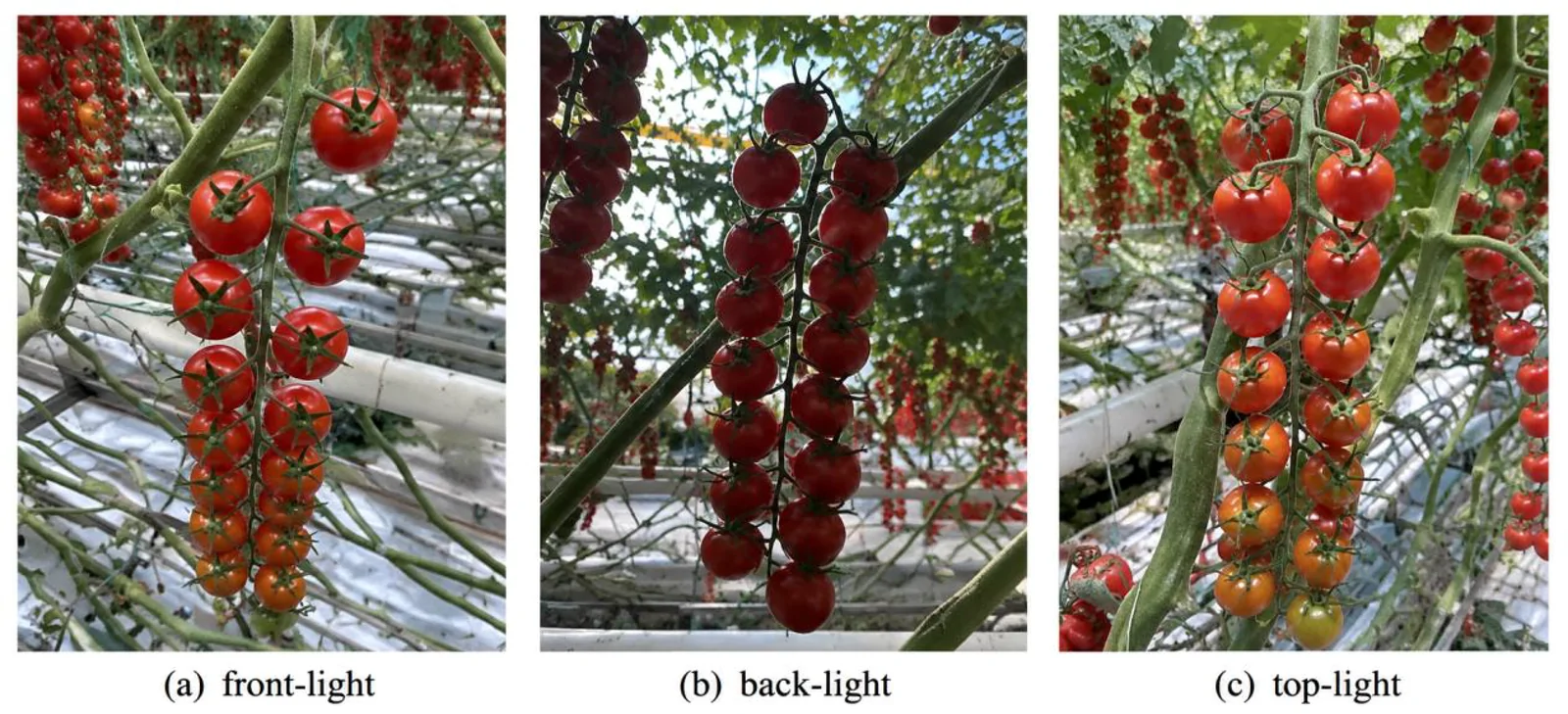
Representative images from the publicly available tomato dataset acquired under different illumination directions. **(a)** Front-light, **(b)** back-light, **(c)** top-light.

The dataset also covered variations in acquisition time and illumination direction, providing visual samples under different temporal and lighting conditions. Representative examples are shown in **Figure 18**.

**Figure 17:**
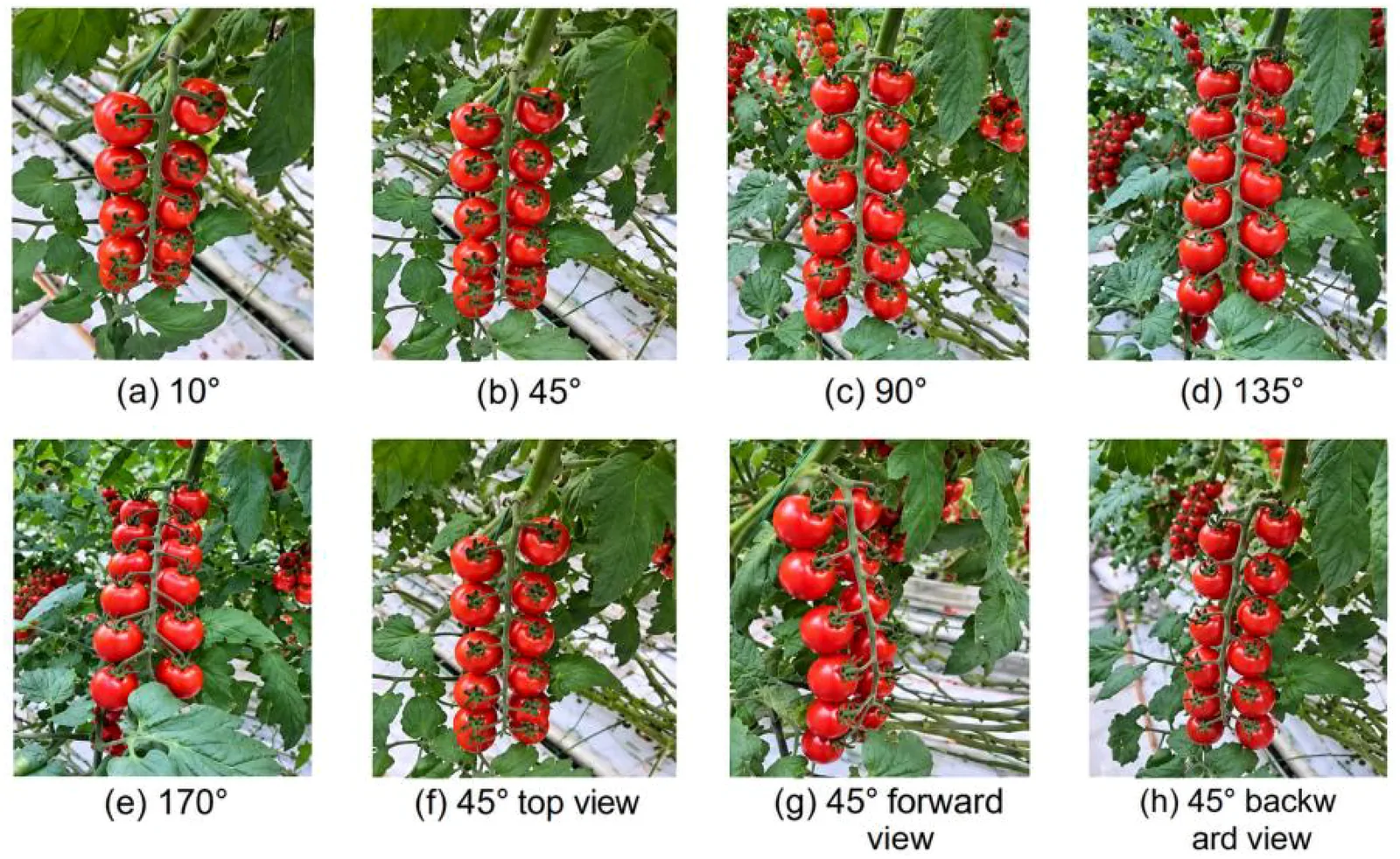
Representative images from different viewpoints and shooting directions in the publicly available tomato dataset. **(a)** 10°, **(b)** 45°, **(c)** 90°, **(d)** 135°, **(e)** 170°, **(f)** 45° top view, **(g)** 45° forward view, **(h)** 45° backward view.

**Figure 18:**
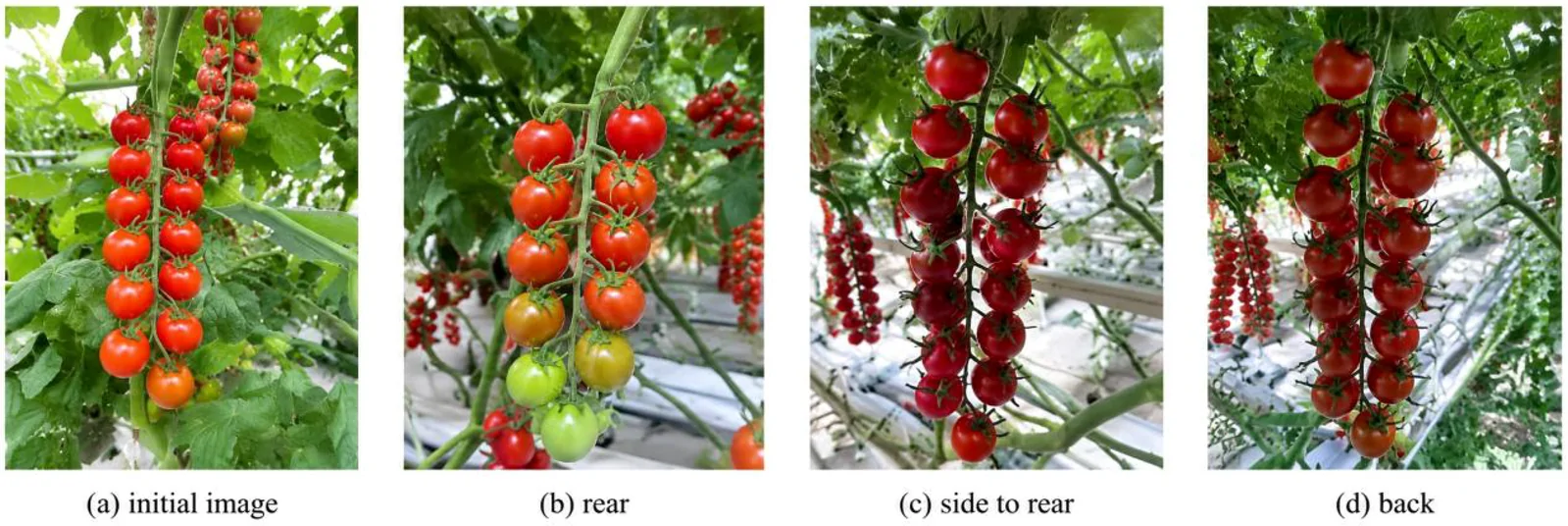
Representative images from the publicly available tomato dataset acquired at different times of day. **(a)** Morning, **(b)** noon, **(c)** afternoon, **(d)** dusk.

According to the published dataset description, the dataset was established through image acquisition, image screening, annotation, and dataset partitioning.The tomato detection dataset consisted of 3665 original images obtained from a publicly available tomato image dataset. An approximately 8:1:1 partition was adopted, resulting in 2932 training images, 367 validation images, and 366 test images. To reduce the risk of information leakage, the original images were partitioned into mutually exclusive training, validation, and test subsets before data augmentation.Data augmentation was subsequently applied only to the training subset, whereas the validation and test subsets contained only original, non-augmented images.

The detailed distribution is summarized in **Table 7**. The original dataset contained 3665 images in total, and the distributions of the annotation categories, illumination conditions, and occlusion conditions are summarized in **Table 7**.

**Table 7:** Distribution of the original dataset according to annotation category, illumination, and occlusion conditions.

Attribute	Category	No. of images	Proportion (%)
Annotation category	Mature	2513	68.6
	Raw	1156	31.5
	Covered mature	2298	62.7
Lighting	Normal/natural light	1558	42.5
	Weak light	1019	27.8
	Strong light	1088	29.7
Occlusion	No occlusion	1297	35.4
	Approximately 30% leaf occlusion	1206	32.9
	Fruit-overlap occlusion	1162	31.7

Mature, raw, and covered-mature were treated as mutually exclusive operational detection labels. Covered-mature does not represent an independent biological maturity state.

Partial leaf occlusion was explicitly included in the dataset rather than removed during data preparation. The original dataset contained 1,206 images acquired under approximately 30% leaf occlusion. When the maturity of a partially visible cluster remained identifiable, the image was retained and annotated according to the original dataset protocol. In particular, a mature cluster partially occluded by leaves or branches was assigned the covered-mature label. These samples enabled the detector to learn discriminative features from the remaining visible color, contour, texture, and cluster-structure information.

Before being input into the detection network, all images were resized to 640 × 640 pixels.

### 6.2 Model selection and improvement

In this study, YOLOv8n was selected as the baseline model. This model demonstrates excellent performance in real-time inference, model compactness, and deployment friendliness, making it particularly suitable for edge-computing devices (Zhang X. et al., 2024). Compared with previous versions such as YOLOv5 and YOLOv7, YOLOv8n adopts a more efficient backbone network and decoupled head structure (Liu et al., 2024; Yao et al., 2026), which significantly improves detection accuracy and training convergence speed while maintaining extremely low computational cost and parameter count (Carbone et al., 2025). Its training process integrates advanced dynamic label assignment strategies and data augmentation methods, effectively enhancing the model’s generalization capability in complex scenes. Additionally, YOLOv8n provides a comprehensive export toolchain and optimized TensorRT support, facilitating seamless transition from laboratory validation to real-world edge deployment (Li et al., 2025). After a comprehensive comparison with current mainstream YOLO series models, YOLOv8n was selected as the baseline because it provides a suitable compromise between detection capability, architectural extensibility, and deployment compatibility for the proposed modifications (Kim et al., 2023; Hou et al., 2025; Singh et al., 2026). Therefore, it was chosen as the foundational framework for this study.

#### 6.2.1 Network improvement strategy

Partial occlusion in tomato clusters can affect detection in two stages. At the feature-extraction and fusion stage, only a portion of the target remains visible, weakening the transmission of fine spatial details and high-level semantic information across scales. At the bounding-box regression stage, the visible target boundary may no longer coincide with the complete physical extent of the cluster, making direct localization more sensitive to ambiguous or incomplete boundary cues. Therefore, the proposed modifications were designed to address these two complementary effects: BiFPN enhances bidirectional cross-scale feature interaction to compensate for incomplete visual information, whereas WIoU improves the optimization of difficult bounding-box regression cases without introducing additional inference branches.

During inference, partially leaf-occluded clusters are processed directly by the YOLOv8n-BiFPN-WIoU detector. BiFPN strengthens bidirectional information exchange among the P3, P4, and P5 feature levels so that the remaining local details and high-level semantic information can complement each other when part of a cluster is hidden. WIoU is applied during training to reduce the sensitivity of bounding-box regression to incomplete or ambiguous boundaries. The method does not reconstruct completely invisible fruits; it detects the cluster from the visible evidence that remains available in the RGB image.

The attention configuration evaluated in ablation groups A2, A4, and A6 was adopted from Ren et al (Qin et al., 2025). It consists of a Channel-embedded Position Attention Module (CePAM) and a Large-Kernel Convolutional Block Attention Module (LKCBAM). CePAM was applied to the P3 shallow feature layer to preserve positional and fine-grained spatial information, thereby improving the representation of small and partially occluded tomato targets. LKCBAM was applied to the P4 feature layer to enlarge the effective receptive field and capture longer-range feature dependencies. The two modules were jointly evaluated as the CePAM–LKCBAM attention configuration. However, this configuration was used only as an ablation candidate and was not retained in the final YOLOv8n-BiFPN-WIoU detector.

According to the overall detection performance and deployment requirements, the combination of BiFPN and WIoU was ultimately selected as the final proposed architecture, hereafter referred to as YOLOv8n-BiFPN-WIoU.The CePAM–LKCBAM attention configuration was retained only as an ablation candidate in configurations A2, A4, and A6 and was not included in the final A5 detector. Although A6 achieved a slightly higher mAP@0.5 than A5 (92.450% vs. 92.338%), the difference was only 0.112 percentage points. In contrast, A5 achieved a higher Recall (89.377% vs. 87.715%) and a slightly higher mAP@0.5:0.95 (71.368% vs. 71.246%). Because missed cluster detections directly prevent the target from entering the subsequent three-dimensional localization and manipulation pipeline, Recall was considered more directly related to downstream harvesting reliability. The higher mAP@0.5:0.95 of A5 further indicates that its localization performance was not sacrificed for the gain in Recall. Therefore, A5 was selected as the final detector based on the combined consideration of missed-detection risk and localization robustness rather than the highest value of a single detection metric.

To improve multi-scale feature representation and bounding-box localization while preserving the lightweight characteristics of YOLOv8n, two principal modifications were introduced into the baseline detector. First, the original neck feature-fusion structure was replaced with a BiFPN-based bidirectional weighted feature-fusion network. Within the BiFPN neck, BiFPN_Concat2 serves as the weighted fusion operator at the corresponding multi-input fusion nodes rather than as an independent network module. It assigns learnable normalized weights to input feature maps before fusion, thereby strengthening information interaction among the P3/8, P4/16, and P5/32 feature levels (Song et al., 2025).

Second, the original bounding-box regression loss was replaced with WIoU during training. WIoU is applied to the bounding-box regression branch after the detection head and affects only the optimization process; therefore, it does not introduce additional layers or computational branches into the forward inference graph. The complete architecture and the positions of the retained modifications are shown in **Figure 19**.

**Figure 19:**
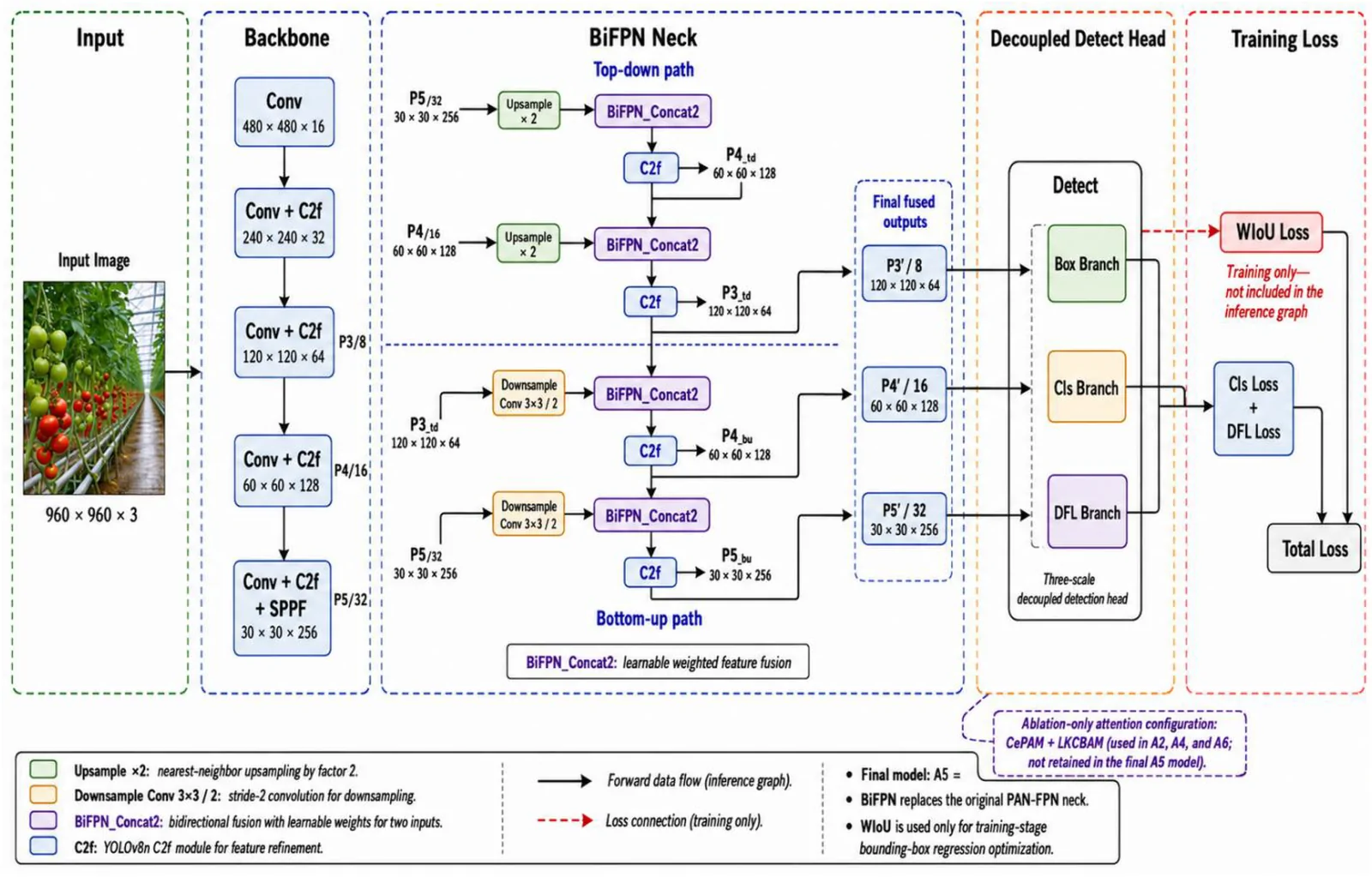
YOLOv8n structural framework. The input resolution of the final detector is 640 × 640 pixels. The YOLOv8n backbone extracts three principal feature levels, P3/8, P4/16, and P5/32, corresponding to spatial resolutions of 80 × 80, 40 × 40, and 20 × 20, respectively. The original neck is replaced by a BiFPN-based bidirectional feature-fusion structure. BiFPN_Concat2 is implemented at the corresponding multi-input fusion nodes to perform learnable weighted feature aggregation. The fused multi-scale features are subsequently delivered to the decoupled detection head. During training, WIoU is applied to the bounding-box regression branch.

The detailed configuration of the final YOLOv8n-BiFPN-WIoU detector is summarized in **Table 8**. With an input resolution of 640 × 640 pixels, the YOLOv8n backbone generates three principal feature levels, P3/8, P4/16, and P5/32, with spatial resolutions of 80 × 80, 40 × 40, and 20 × 20 and channel dimensions of 64, 128, and 256, respectively. The BiFPN neck performs bidirectional weighted feature fusion across these three scales. BiFPN_Concat2 is used at the four multi-input fusion nodes, producing intermediate feature dimensions of 384, 192, 192, and 384 channels, which are subsequently refined by C2f modules to generate the final P3′/8, P4′/16, and P5′/32 features with 64, 128, and 256 channels, respectively. These three fused feature maps are supplied to the decoupled Detect head for multi-scale prediction.

**Table 8:** Detailed network configuration of the proposed YOLOv8n-BiFPN-WIoU detector.

Stage	Output scale	Output channels	Main modification
Input	640 × 640	3	–
P3/8	80×80	64	YOLOv8n backbone
P4/16	40 × 40	128	YOLOv8n backbone
P5/32	20 × 20	256	YOLOv8n backbone+ SPPF
P3′/8	80×80	64	BiFPN_Concat2
P4′/16	40 × 40	128	BiFPN_Concat2
P5′/32	20 × 20	256	BiFPN_Concat2
Detect	Three scales	64/128/256	Decoupled head + WIoU*

*WIoU is used only during training for bounding-box regression.

#### 6.2.2 BiFPN weighted feature fusion module

Recent tomato-detection studies have demonstrated that lightweight feature enhancement and multi-scale representation can improve the detection of small, overlapping, and partially occluded tomato targets in complex greenhouse environments (Rong et al., 2023; Yang et al., 2023; Huang et al., 2024; Huang et al., 2025; Qin et al., 2025; Zhao et al., 2025). These challenges are particularly evident in clustered tomato scenes, where variations in fruit scale and visibility may weaken the effective transmission of target features across different network levels. To strengthen cross-scale feature aggregation, a BiFPN-based weighted bidirectional feature-fusion structure was therefore introduced into the YOLOv8n neck.

To solve this problem, the BiFPN structure was introduced into the YOLOv8n neck. Unlike the traditional PAN-FPN structure, BiFPN constructs a bidirectional feature fusion path and introduces learnable weights to balance the contribution of different input features. At each BiFPN_Concat2 node, the input feature maps were first resized to the same spatial resolution and then fused using normalized non-negative learnable weights, as defined in Equation 15.

(15) $$F_{out}=Concat(\frac{w_{1}F_{1}}{\epsilon +\sum_{j=1}^{n}w_{j}},\frac{w_{2}F_{2}}{\epsilon +\sum_{j=1}^{n}w_{j}},\frac{w_{n}F_{n}}{\epsilon +\sum_{j=1}^{n}w_{j}}),w_{j}=ReLU({\hat{w}}_{j})$$

where ${\text{F}}_{\text{j}}$ denotes the j-th aligned input feature map, $\hat{w_{j}}$ is a trainable scalar, $w_{j}$ is the corresponding non-negative fusion weight, and ϵ is a small constant used to avoid numerical instability. The weighted features are concatenated along the channel dimension and subsequently refined by the C2f module.

In this way, the network can automatically strengthen effective feature layers and suppress less informative feature responses during training (Zhang et al., 2023).

In the proposed network, the P3 feature layer provides more detailed spatial information, which is useful for detecting small tomato targets; the P4 feature layer contains medium-scale semantic information; and the P5 feature layer provides high-level semantic features. Through repeated top-down and bottom-up weighted fusion, the BiFPN module enhances the model’s ability to detect tomato clusters with different sizes, occlusion levels, and spatial distributions.

According to the network configuration, the backbone generates three feature maps, denoted by ${\text{P}}_{3},{\text{P}}_{4}$,and ${\text{P}}_{5}$​, with spatial resolutions of 80×80,40×40,and 20×20 and channel dimensions of 64, 128, and 256, respectively. The original Concat operations in the YOLOv8n neck were replaced by four BiFPN_Concat2 operators. For two spatially aligned input feature maps ${\text{F}}_{\text{a}}$ and ${\text{F}}_{\text{b}}$ ​, the weighted concatenation is expressed in Equation 16.

(16) $$ℬ({\text{F}}_{\text{a}},{\text{F}}_{\text{b}})=\text{Concat}({\text{λ}}_{\text{a}}{\text{F}}_{\text{a}},{\text{λ}}_{\text{b}}{\text{F}}_{\text{b}}),\;{\text{λ}}_{\text{k}}=\frac{{\bar{\text{w}}}_{\text{k}}}{\text{ϵ}+{\bar{\text{w}}}_{\text{a}}+{\bar{\text{w}}}_{\text{b}}},\text{ k}\in \{\text{a},\text{b}\}$$

where ${\bar{\text{w}}}_{\text{a}}$ and ${\bar{\text{w}}}_{\text{b}}$ denote the effective learnable fusion weights, ${\text{λ}}_{\text{a}}$ and ${\text{λ}}_{\text{b}}$ are their normalized values, and ϵ is a small constant introduced to avoid numerical instability. Unlike conventional concatenation, this operation adaptively scales the contribution of each input branch before channel-wise concatenation. The resulting top-down and bottom-up feature-fusion paths are expressed in Equation 17.

(17) $$\begin{array}{c} {\text{P}}_{4}^{\text{td}}={\text{Φ}}_{128}[ℬ(U({\text{P}}_{5}),{\text{P}}_{4})],\\ {\text{P}}_{3}^{\text{out}}={\text{Φ}}_{64}[ℬ(U({\text{P}}_{4}^{\text{td}}),{\text{P}}_{3})],\\ {\text{P}}_{4}^{\text{out}}={\text{Φ}}_{128}[ℬ(D({\text{P}}_{3}^{\text{out}}),{\text{P}}_{4}^{\text{td}})],\\ {\text{P}}_{5}^{\text{out}}={\text{Φ}}_{256}[ℬ(D({\text{P}}_{4}^{\text{out}}),{\text{P}}_{5})]. \end{array}$$

where U(·) denotes nearest-neighbor upsampling by a factor of 2, D(·)  denotes downsampling using a 3×3 convolution with a stride of 2, and ${\text{Φ}}_{\text{c}}(\cdot )$ denotes the subsequent C2f refinement operation with cc output channels. The top-down path produces ${\text{P}}_{4}^{\text{td}}$ and ${\text{P}}_{3}^{\text{out}}$, whereas the bottom-up path produces ${\text{P}}_{4}^{\text{out}}$ and ${\text{P}}_{5}^{\text{out}}$. Finally, ${\text{P}}_{3}^{\text{out}},{\text{P}}_{4}^{\text{out}},$ and ${\text{P}}_{5}^{\text{out}}$ are supplied to the decoupled Detect head for three-scale prediction. The corresponding network structure is shown in **Figure 20**.

**Figure 20:**
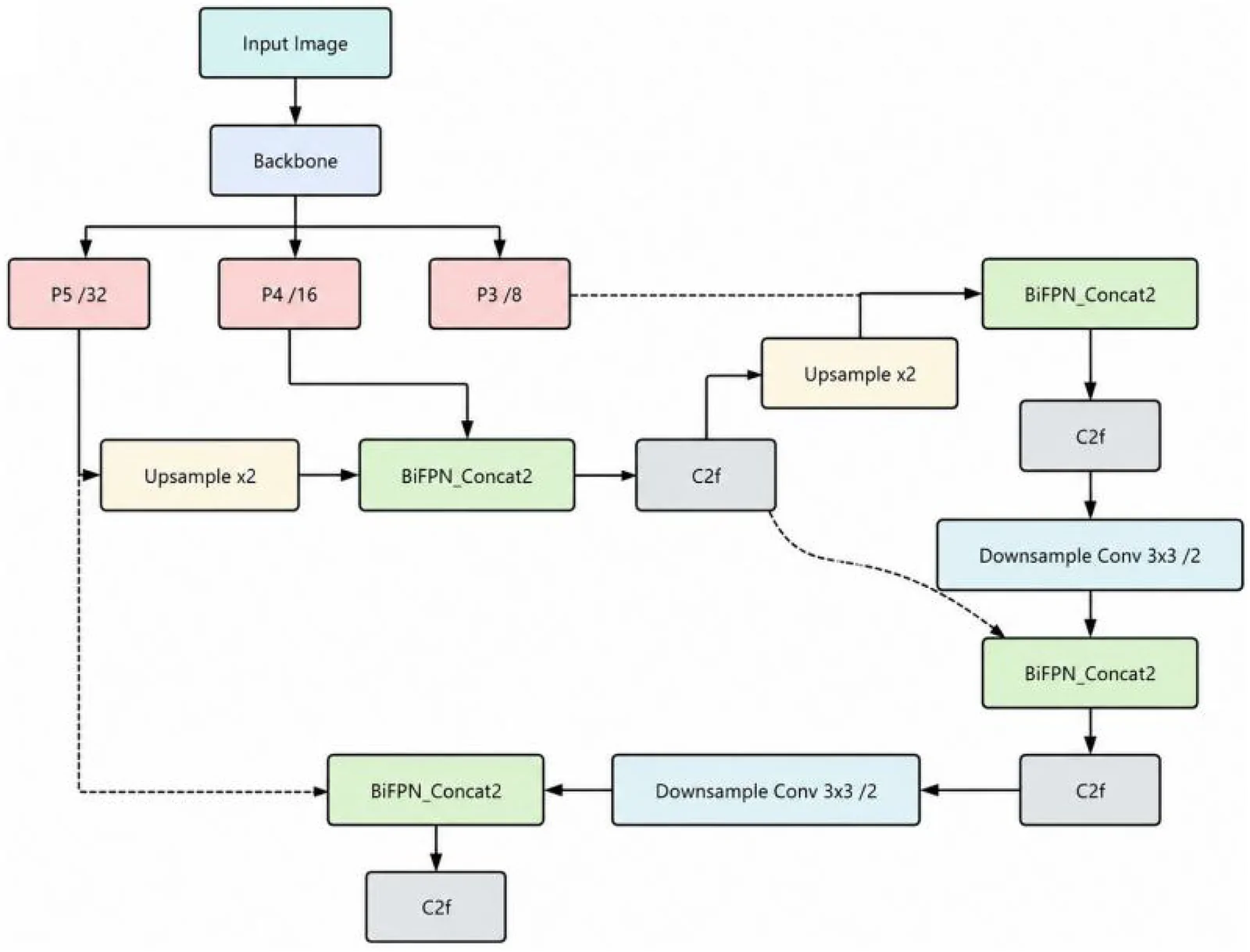
Detailed structure of the BiFPN neck and BiFPN_Concat2 weighted feature-fusion nodes.

Compared with the simple concatenation strategy used in the original YOLOv8n neck, the normalized learnable weighting defined in Eqs. (20) and (21) enables the network to adaptively regulate the contribution of different input branches during multi-scale feature fusion. This design retains the original three-scale detection framework while strengthening cross-layer feature transmission and semantic compensation (Wang T. et al., 2023). For partially occluded tomato clusters, the visible boundary and texture cues retained in $P_{3}$ can be complemented by the higher-level semantic information contained in $P_{4}$ and $P_{5}$. The resulting feature maps therefore provide more complete representations for the detection head under complex greenhouse backgrounds, leaf occlusion, fruit overlap, and target-scale variation (Zhang J. et al., 2024). Related improved YOLOv5-based methods have also demonstrated the value of multi-scale feature enhancement for greenhouse tomato detection and pose classification under comparable visual conditions (Zheng et al., 2024).

**Figure 21:**
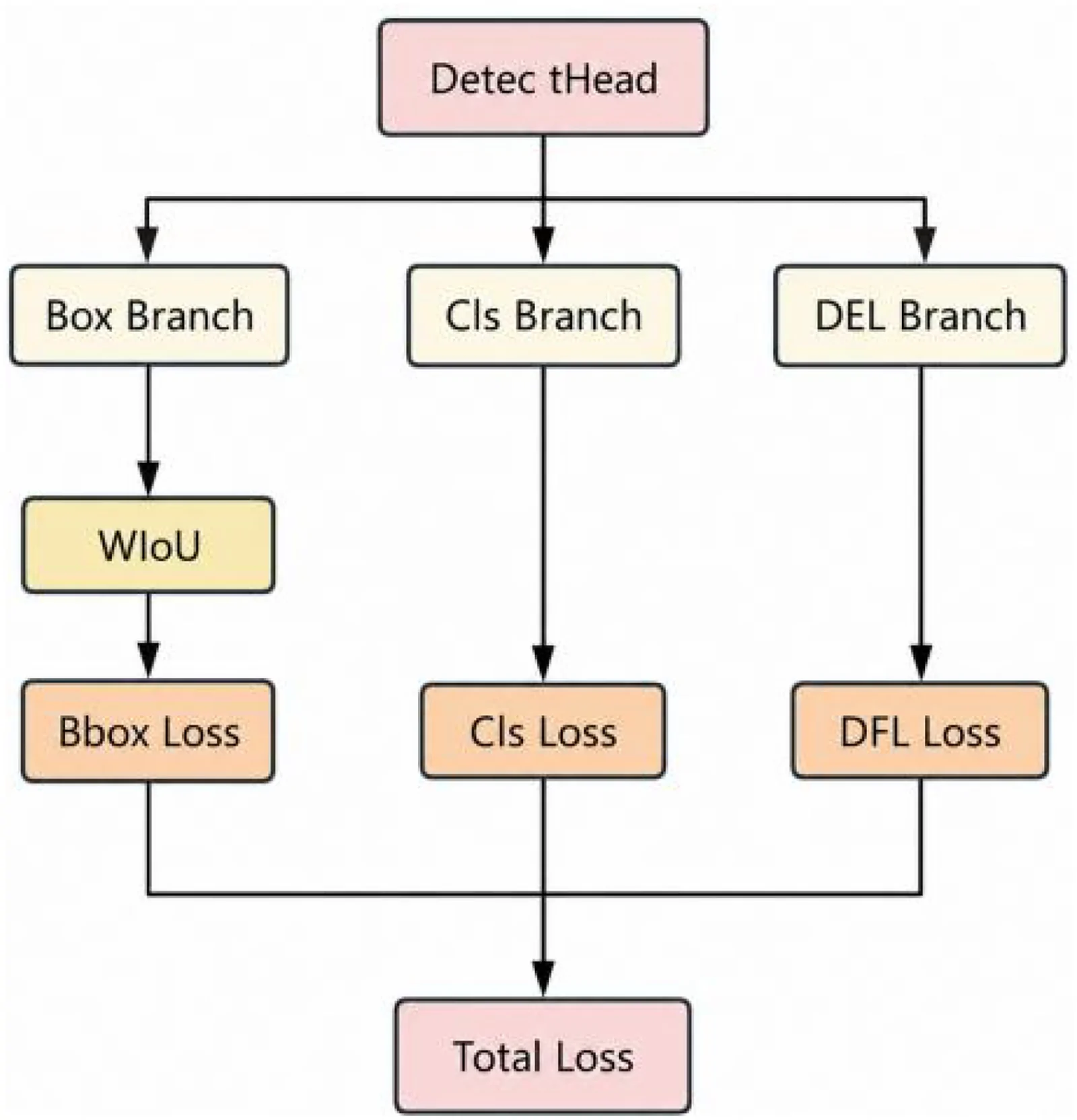
Integration of WIoU into the bounding-box regression branch during model training.

In the A5_BiFPN_WIoU network, WIoU is adopted as the bounding-box regression loss in the regression optimization component. As illustrated in the loss optimization pipeline, the Detect Head is divided into three branches, namely the Box Branch, Cls Branch, and DFL Branch, which correspond to bounding-box regression, category classification, and distribution focal learning, respectively. In the Box Branch, WIoU is introduced to optimize the Bbox Loss, as illustrated in Equation 21. Compared with methods that rely only on conventional IoU constraints, WIoU places greater emphasis on the dynamic modeling of sample quality and regression difficulty, enabling the training process to assign gradients more specifically to high-quality samples, low-quality samples, and difficult samples. This loss module is located after the Detect Head and participates only in the training stage without altering the forward inference structure. Its main function is to improve the stability and convergence efficiency of localization regression, thereby further enhancing the matching quality between predicted bounding boxes and ground-truth boxes. Together with the Cls Loss and DFL Loss, the optimized Bbox Loss constitutes the Total Loss used for model training.

### 6.3 Experiments and results

#### 6.3.1 Experimental environment and evaluation metrics

Model training, ablation experiments, comparative experiments, and offline evaluation were conducted on a desktop workstation equipped with an Intel Core i5-12600KF processor, 16 GB RAM, and an NVIDIA GeForce RTX 4060 GPU with 8 GB of memory. The workstation ran 64-bit Windows 11, with Python 3.8.21, PyTorch 1.13.1, torchvision 0.14.1, and CUDA 11.7. The AdamW optimizer was used for model training. All models were trained and evaluated using an input resolution of 640 × 640 pixels to ensure consistency across the baseline, ablation, and comparative experiments. The principal training parameters are summarized in **Table 9**.

**Table 9:** Training configuration parameters.

Parameter	Value
Input Image Size	640×640
Training Epochs	150 epochs
Number of Data Loading Workers	8
Initial Learning Rate (lr0)	0.001
Final Learning Rate Coefficient (lrf)	0.01
Weight Decay Coefficient	0.0005
Optimizer	AdamW
Early Stopping Patience	100 epochs
Pretrained Weights	True
AMP	False
Batch Size	12

After model development and offline evaluation, the final YOLOv8n-BiFPN-WIoU model was exported and deployed on the NVIDIA Jetson Nano installed on the harvesting robot for edge inference. Therefore, the RTX 4060 workstation served as the model-development, training, and offline-evaluation platform, whereas the Jetson Nano served exclusively as the edge-deployment and online-inference platform.The same input resolution was used for the baseline, ablation, and comparative experiments to ensure a consistent evaluation protocol.

To evaluate the practical edge-deployment performance of the proposed YOLOv8n-BiFPN-WIoU model, a dedicated inference experiment was conducted on the NVIDIA Jetson Nano platform. The trained PyTorch model was first exported to ONNX and subsequently converted to a TensorRT inference engine. All tests used an input resolution of 640 × 640 pixels and a batch size of 1.

The Jetson Nano was operated in the 10-W MAXN mode.NVIDIA specifies MAXN as the 10-W mode (mode ID 0) for Jetson Nano. The Jetson Nano was operated in the 10-W MAXN mode using JetPack 4.6.1. The deployment environment consisted of CUDA 10.2, TensorRT 8.2.1, and L4T 32.7.1.

The TensorRT engine used FP16 precision. Before timing, 50 warm-up frames were processed to stabilize GPU execution. Latency was then averaged over 1,000 consecutive test frames. The reported end-to-end latency included image preprocessing, TensorRT inference, and non-maximum suppression (NMS). The PyTorch implementation and the TensorRT-optimized implementation were evaluated using the same input images and timing protocol.

The primary detection metrics were Precision, Recall, F1-score, mAP@0.5, and mAP@0.5:0.95, with model parameters, GFLOPs, model size, and edge inference speed used to characterize deployment cost. Recall was emphasized because missed cluster detections directly prevent subsequent localization and harvesting. The evaluation metrics are defined by Equations 18–22.

(18) $$\begin{array}{c} \text{P}=\frac{{\text{T}}_{\text{P}}}{{\text{T}}_{\text{P}}+{\text{F}}_{\text{P}}} \end{array}$$

(19) $$\text{R}=\frac{{\text{T}}_{\text{P}}}{{\text{T}}_{\text{P}}+{\text{F}}_{\text{N}}}$$

(20) $$\text{AP}=\int_{0}^{1}{\text{P}}_{\text{r}}(\text{R})\text{dR}$$

(21) $$\text{mAP}=\frac{\sum_{\text{i}=1}^{\text{n}}\text{AP}(\text{n})}{\text{m}}$$

(22) $$\text{F}1\text{ Score}=2\cdot \frac{\text{Precision}\cdot \text{Recall}}{\text{Precision}+\text{Recall}}$$

All reported detection metrics were calculated using the independent test subset containing only original, non-augmented images. The validation subset was used only for model selection and training monitoring and was not used for final performance reporting.

#### 6.3.2 Ablation experiments

This study conducted ablation experiments using YOLOv8n as the baseline model to evaluate the individual and combined effects of the BiFPN-based feature-fusion modification, the attention-module configuration, and the WIoU-based bounding-box regression loss. Among the evaluated configurations, A5, corresponding to YOLOv8n-BiFPN-WIoU, was selected as the final detection model for the proposed harvesting system based on the overall ablation results. The results are shown in **Table 10**.

**Table 10:** Ablation study results.

ID	Model bariant	Lightweight feature fusion	Attention module	Bounding box loss function	Precision	Recall	mAP@0.5	mAP@0.5:0.95
A0	YOLOv8n	×	×	CIoU	86.391	86.598	87.751	65.693
A1	YOLOv8n-BiFPN	✓	×	CIoU	90.534	88.934	92.186	71.581
A2	YOLOv8n-Att	×	✓	CIoU	91.274	87.957	92.05	72.095
A3	YOLOv8n-WIoU	×	×	WIoU	91.072	87.645	91.42	71.963
A4	YOLOv8n-BiFPN-Att	✓	✓	CIoU	88.931	87.568	91.761	70.352
A5	YOLOv8n-BiFPN-WIoU	✓	×	WIoU	88.534	89.377	92.338	71.368
A6	YOLOv8n-Att-WIoU	×	✓	WIoU	90.816	87.715	92.45	71.246

“Att” denotes the joint CePAM–LKCBAM attention configuration adopted from Ren et al. (2025). In this configuration, CePAM was applied to the P3 shallow feature layer, whereas LKCBAM was applied to the P4 feature layer. The attention configuration was evaluated only in A2, A4, and A6 and was not retained in the final A5 detector. BiFPN_Concat2 is the weighted feature-fusion operator embedded within the BiFPN neck and was therefore not treated as an independent ablation factor. A5 was selected as the final proposed detector.

According to **Table 10**, the original YOLOv8n baseline model already demonstrates a certain detection capability in the greenhouse tomato target recognition task. However, under complex greenhouse conditions, problems such as insufficient feature fusion and limited localization accuracy for occluded targets still exist, indicating that the model has considerable room for improvement. After introducing the lightweight feature fusion module, Precision, Recall, mAP@0.5, and mAP@0.5:0.95 all increased after introducing the BiFPN-based feature-fusion module. In particular, mAP@0.5 increases from 87.751% to 92.186%, indicating that this module can effectively enhance information interaction among features at different scales and improve the model’s ability to detect small targets, overlapping tomatoes, and occluded tomatoes. After adding the attention module, Precision increases to 91.274%, and mAP@0.5:0.95 increases to 72.095%, suggesting that the attention mechanism can guide the network to focus more on tomato fruits, fruit stems, and maturity-related regions, thereby suppressing interference from complex backgrounds and improving detection accuracy. After adopting the improved IoU loss function, Precision reaches 91.072%, and mAP@0.5 reaches 91.420%, demonstrating that the improved loss function can optimize the bounding-box regression process and enhance the accuracy of target localization and the stability of training convergence (Fu et al., 2024; Sun, 2024).

The combined experimental results show that different improved modules have a certain complementary effect. Among them, the combination of the lightweight feature fusion module and the improved loss function achieves the highest Recall of 89.377% among all experimental groups, indicating that this combination can effectively reduce missed detections of tomato targets. The combination of the attention module and the improved loss function achieves the highest mAP@0.5 of 92.450%, which is 4.699 percentage points higher than that of the baseline model, indicating that this combination achieves the highest mAP@0.5 among the evaluated configurations. The ablation experiments demonstrated the individual effects of the evaluated feature-fusion, attention, and bounding-box regression strategies. Based on the downstream harvesting requirements, YOLOv8n-BiFPN-WIoU (A5) was selected as the final detector because of its higher Recall and comparable localization performance.A6 achieved the highest mAP@0.5 (92.450%), whereas A5 achieved a higher Recall (89.377% vs. 87.715%) and a slightly higher mAP@0.5:0.95 (71.368% vs. 71.246%). The mAP@0.5 difference between the two configurations was only 0.112 percentage points. Because missed detections directly prevent subsequent localization and harvesting execution, the higher Recall of A5 was prioritized when selecting the final detector.

#### 6.3.3 Comparative experiments

To provide a controlled comparison with representative object-detection models, YOLOv5n, YOLOv7-tiny, YOLOv8n, YOLOv10n, RT-DETR-l, and the proposed YOLOv8n-BiFPN-WIoU model were evaluated using the same dataset partitions, input resolution, evaluation metrics, and computing platform. All models were trained for 150 epochs and trained and evaluated on the RTX 4060 workstation. The same training-set partition, validation-set partition, test-set partition, data-augmentation strategy, input resolution, batch size, and pretrained-weight setting were used across the compared models to ensure a consistent evaluation protocol. Edge-inference performance was measured separately on the NVIDIA Jetson Nano using the same benchmarking protocol (Urrea, 2025; Fan and Chai, 2026). Precision, Recall, F1-score, mAP@0.5, mAP@0.5:0.95, parameter count, GFLOPs, model size, and Jetson Nano inference speed were used for comparison, as summarized in **Table 11**.

**Table 11:** Performance comparison of different models.

Model	P (%)	R (%)	F1 (%)	mAP@0.5 (%)	mAP@0.5:0.95 (%)	Parameters (M)/GFLOPs	Model size (MB)/Jetson Nano FPS
YOLOv5n	89.257	86.499	87.856	89.249	65.494	1.9 M/10.1	4.1 MB/22.6
YOLOv7-tiny	88.760	87.230	87.988	89.760	66.770	6.2 M/29.7	12.3 MB/15.4
YOLOv8n	86.391	86.598	86.494	87.751	65.693	3.2 M/19.6	6.3 MB/22.8
YOLOv10n	87.854	84.645	86.220	87.586	67.173	2.3 M/15.1	5.7 MB/23.6
RT-DETR-l	73.801	80.440	76.978	78.984	52.843	32.0 M/243.0	65.8 MB/5.3
YOLOv8n-BiFPN-WIoU	88.534	89.377	88.954	92.338	71.368	3.6 M/22.4	7.2 MB/21.1

All models were evaluated at 640 × 640 resolution and trained for 150 epochs. F1 was calculated from Precision and Recall. YOLO26n was not considered in the original comparative experiment and was therefore not evaluated in the present study. Accordingly, the conclusions of the comparative analysis are limited to the models listed in **Table 11**. The absence of a YOLO26n comparison is acknowledged as a limitation of the current study.

As shown in **Table 11**, the proposed YOLOv8n-BiFPN-WIoU model achieved the highest Recall, F1-score, mAP@0.5, and mAP@0.5:0.95 among the evaluated detectors. Compared with the baseline YOLOv8n, Recall increased from 86.598% to 89.377%, F1-score increased from 86.494% to 88.954%, mAP@0.5 increased from 87.751% to 92.338%, and mAP@0.5:0.95 increased from 65.693% to 71.368%. Although YOLOv5n achieved a slightly higher Precision and YOLOv10n showed a slightly higher edge inference speed, the proposed model provided a better overall balance between detection accuracy, missed-detection reduction, localization quality, and edge-computing efficiency.

The introduction of the BiFPN neck resulted in only a moderate increase in model complexity compared with the baseline YOLOv8n.At an input resolution of 640 × 640 and a batch size of 1, the proposed model contained approximately 3.6 M parameters, required 22.4 GFLOPs, had a model size of approximately 7.2 MB, and achieved an average inference speed of 21.1 FPS on the Jetson Nano. This indicates that the accuracy improvement was achieved without substantially compromising the lightweight and real-time characteristics required for greenhouse robotic harvesting.

#### 6.3.4 Comparison diagram of visualization effects

As illustrated in **Figure 22**, the evaluated models showed different responses to occlusion, target overlap, and reduced target scale. The proposed YOLOv8n-BiFPN-WIoU detector maintained more consistent target coverage and localization under severe occlusion and distant small-target conditions, whereas RT-DETR-l and YOLOv10n showed more incomplete or low-confidence predictions in some challenging regions. These qualitative observations are consistent with the quantitative improvements in Recall and mAP reported in **Table 11**.

**Figure 22:**
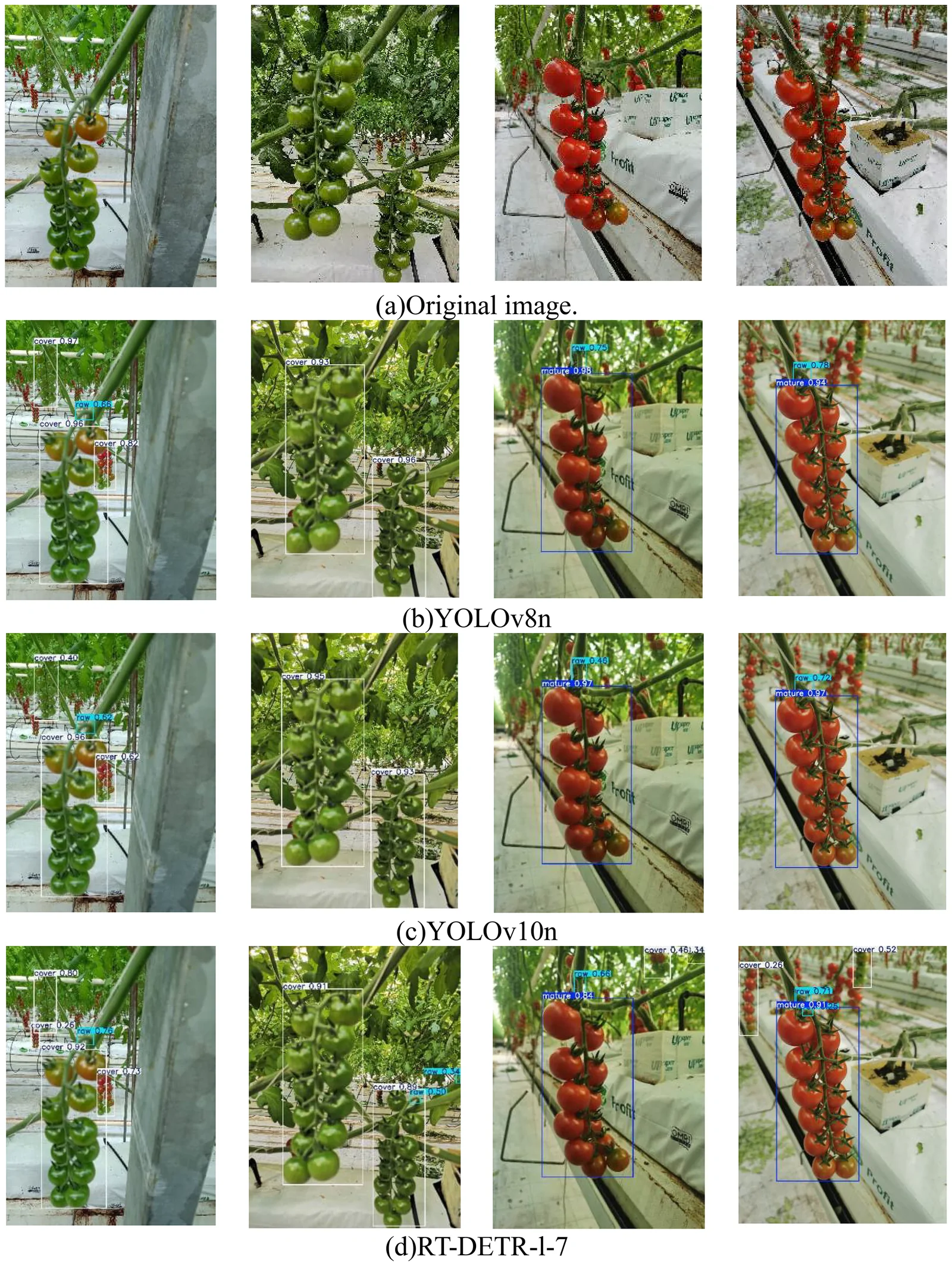
Comparison of visualization effects. **(a)** Original images; **(b)** YOLOv8n; **(c)** YOLOv10n; **(d)** RT-DETR-L.

## 7 Redundant design and fault self-diagnosis & self-recovery functions

### 7.1 Power supply and redundant design of key components

Power redundancy was treated separately from harvesting-system reliability, with the onboard battery configuration described in Section 2.2.

Redundancy was introduced at the controller and pneumatic-actuation levels. A backup STM32 controller was reserved for core control functions, while a backup pneumatic circuit was provided for the cutting system in case of insufficient pressure or primary-valve failure.

### 7.2 Development of fault self-diagnosis and self-recovery functions

A hierarchical fault-management mechanism was implemented at the hardware, communication, and task levels. Recoverable sensor, communication, and ROS-node faults triggered retry or controlled restart, while persistent actuator, communication, or safety faults caused harvesting cancellation and emergency stop. Fault states and recovery actions were recorded for maintenance.

## 8 Experimental testing

Experimental validation was conducted to evaluate the visual perception and whole-system harvesting performance of the proposed greenhouse tomato cluster-harvesting robot under greenhouse conditions. The experiments focused on maturity recognition, harvesting success, fruit damage, false cutting, harvesting performance, and operational reliability.

### 8.1 Experimental variables and control conditions

A single cluster tomato cultivar grown under greenhouse conditions was selected as the experimental material. The tomato clusters were assigned to three mutually exclusive detection labels according to the original dataset annotation scheme: mature, raw, and covered-mature, as shown in Equation 23. Here, the covered-mature label denotes a mature cluster whose visible region is partially occluded by leaves or branches; it is therefore treated as an operational detection label rather than an independent biological maturity state. Accordingly, the three labels were used as mutually exclusive classes during model training and evaluation. In this study, the annotation and recognition unit was the tomato cluster rather than the individual fruit, and each bounding box corresponded to one complete target cluster. Because fruits within the same cluster may naturally exhibit different ripening stages, complete maturity uniformity among all fruits was not required. Instead, the ground-truth maturity label of each cluster was determined according to the dominant maturity stage among the clearly visible fruits. Specifically, the maturity stage represented by the largest number of visible fruits was assigned as the cluster-level label.

In the original dataset annotation scheme, a cluster was labeled as mature when all fruits were red or only one or two fruits showed slight yellow coloration without visible green; otherwise, the cluster was labeled as raw. When a mature cluster was partially occluded by branches or leaves but its maturity remained identifiable, it was assigned the covered-mature detection label.

**Figure 23:**
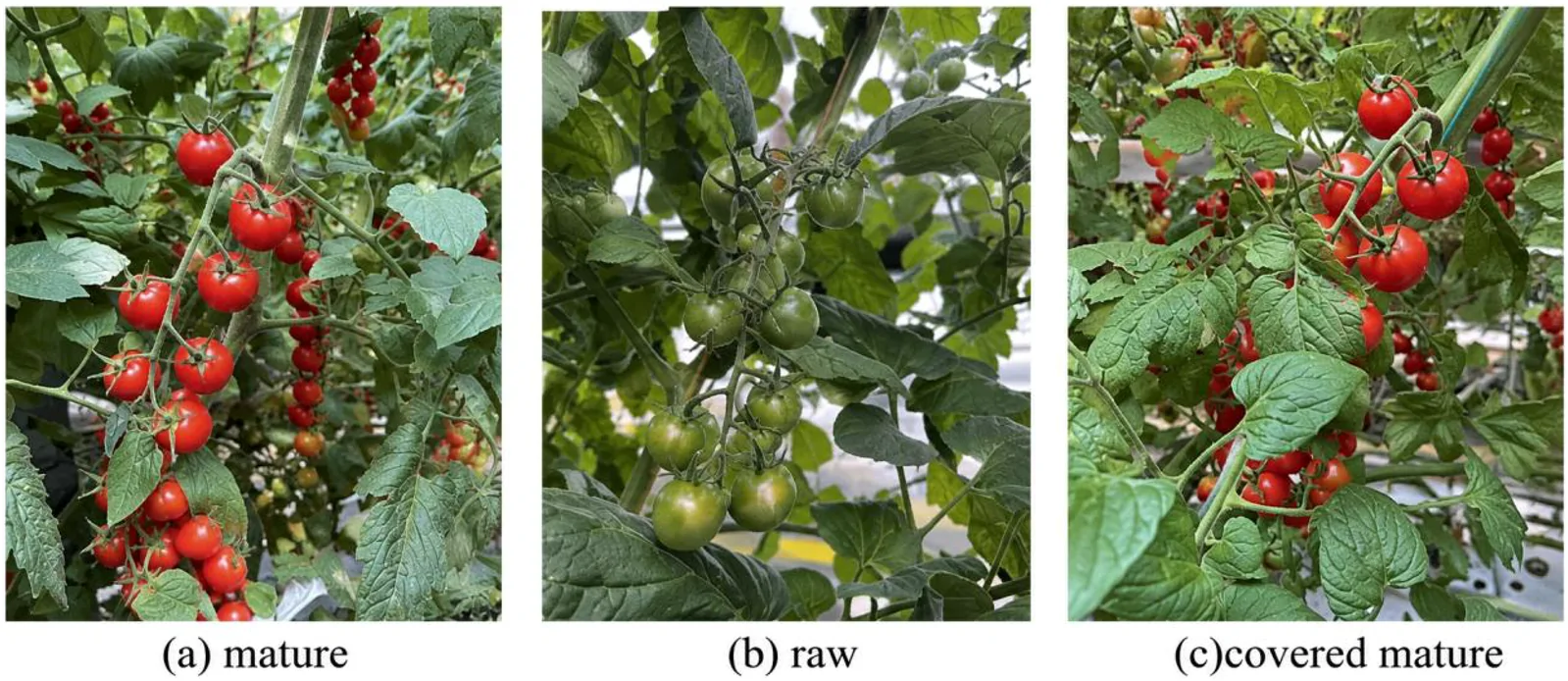
Representative tomato-cluster samples corresponding to the three annotation categories in the publicly available dataset. **(a)** Mature, **(b)** raw, **(c)** covered-mature.

The experiment was conducted in a greenhouse tomato planting base in Dezhou, Shandong. Three typical light environments were set: natural light from 9:00–12:00 (as shown in **Figure 24a**), weak light covered by a sunshade net (as shown in **Figure 24b**), and strong noon light (as shown in **Figure 24c**); three fruit occlusion environments: no occlusion (as shown in **Figure 25a**), 30% leaf occlusion (as shown in **Figure 25b**), and fruit overlapping occlusion (as shown in **Figure 25c**).All robotic harvesting experiments were conducted with the mobile platform operating autonomously along the continuous greenhouse rail network.

**Figure 24:**
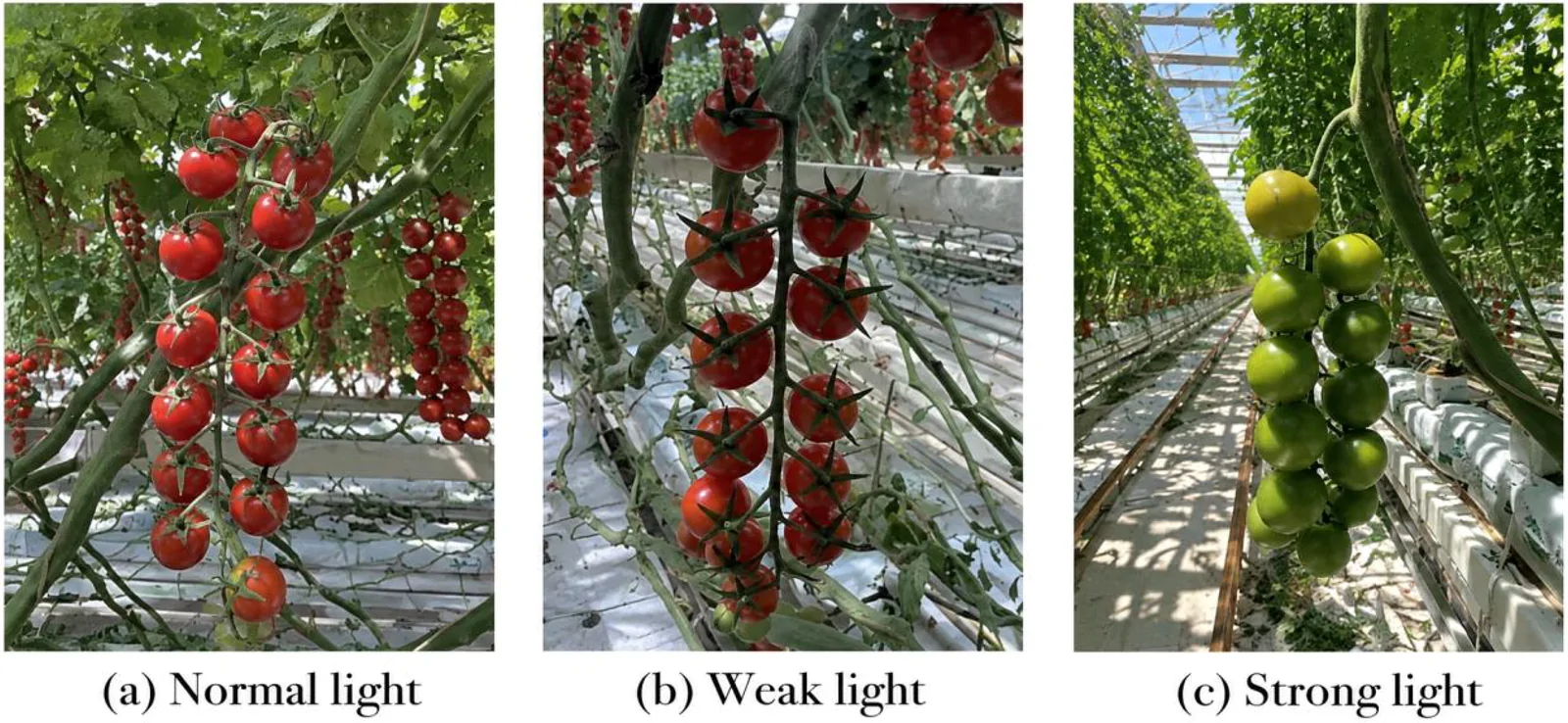
Different light environments. **(a)** Normal light, **(b)** weak light, **(c)** strong light.

**Figure 25:**
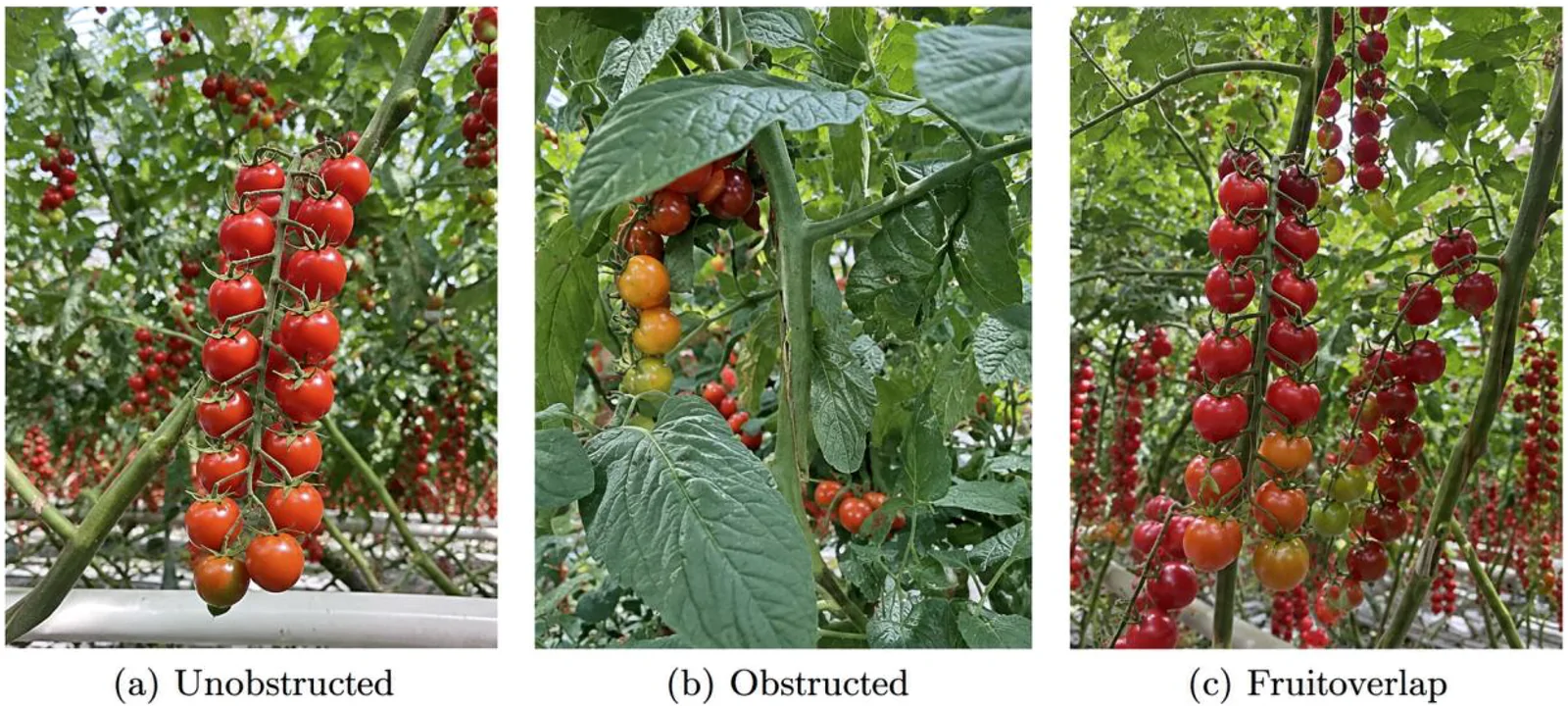
Different occlusion environments. **(a)** Unobstructed, **(b)** obstructed, **(c)** fruit-overlap.

For statistical analysis, one tomato cluster subjected to one complete harvesting operation was defined as one harvesting trial and one statistical unit under real greenhouse conditions. This sample size was used to evaluate harvesting performance, continuous harvesting performance, and operational stability.

The present study focused on the independent evaluation of the proposed robotic harvesting system under real greenhouse conditions, with emphasis on visual perception, harvesting execution, and continuous operational performance. A direct comparison with manual harvesting or conventional mechanical harvesting was not conducted because standardized matched-condition comparative trials were beyond the scope of the present experimental design.

The clamping and positioning errors of the dual-claw structure on the fruit stalk, as well as the cutting angle deviation of the pneumatic scissors, were recorded during harvesting tests. High-speed cameras were used to observe the harvesting process, and thin-film pressure sensors were employed to collect contact force data between the fruit and the gripping claws. These measurements were used to observe the grasping, cutting, and fruit-contact processes during harvesting. In addition, continuous operation tests were conducted for two hours under typical greenhouse rail-guided conditions to assess operational stability and fault occurrence, and operational stability of the robot. For the rail-guided chassis, the station-to-station motion response, stopping accuracy, and positioning error were recorded to assess the reliability of the rail-guided motion control system during repeated harvesting operations.

The proposed robotic system was evaluated in 500 complete greenhouse harvesting trials, with one tomato cluster subjected to one complete harvesting operation considered as one harvesting trial. The completed greenhouse tomato cluster harvesting robot and its integrated harvesting system are shown in **Figure 26**. The system-level performance indicators were defined independently from the object-detection metrics. Recognition-label accuracy was calculated as the number of tomato clusters assigned to the correct recognition label divided by the total number of clusters evaluated. Harvesting success rate was defined as the number of successfully harvested clusters divided by the total number of harvesting attempts. A harvesting attempt was considered successful when the target cluster was detected, approached, grasped, cut, and transferred to the collection unit without dropping or requiring manual intervention. The maturity recognition accuracy was calculated from the greenhouse evaluation samples using the same cluster-level annotation criteria described above. The robot successfully completed 473 of 500 harvesting trials, corresponding to a harvesting success rate of 94.6% (95% CI: 92.3%–96.3%).

The 500 harvesting trials were conducted across the representative maturity, illumination, and occlusion conditions described above. Because detailed condition-wise allocation records were not retained as an independent dataset, condition-specific trial counts are not reported.

Fruit-damage rate was calculated using the number of successfully harvested clusters exhibiting observable harvesting-induced damage as the numerator and the number of successfully harvested clusters subjected to post-harvest damage inspection as the denominator. False-cutting rate was defined as the number of cutting operations deviating from the predefined acceptable stem-cutting region divided by the total number of cutting attempts. These physical harvesting metrics were calculated independently of mAP, Precision, Recall, and other image-based detection metrics.

**Table 12:** Consolidated performance of the proposed harvesting system.

Category	Metric	Final value	Evaluation basis
Detection	mAP@0.5	92.338%	Independent test set
Detection	mAP@0.5:0.95	71.368%	Independent test set
Recognition	Maturity recognition accuracy	92.3%	Greenhouse harvesting trials
Harvesting	Harvesting success rate	94.6% (473/500)	500 harvesting trials
Harvesting	Fruit-damage rate	5.3%	Damaged/inspected clusters
Harvesting	False-cutting rate	6.6%	False cuts/cutting attempts

Detection metrics were obtained from the independent image test set, whereas maturity recognition and harvesting metrics were obtained from whole-system greenhouse experiments. mAP is not equivalent to maturity recognition accuracy. The statistical units, sample sizes, and reporting methods used for the principal experimental outcomes are summarized in **Table 13**.

The whole-system greenhouse experiments were conducted to evaluate maturity recognition, harvesting execution, fruit handling quality, cutting accuracy, harvesting performance, and operational stability under representative greenhouse conditions. The system-level positioning deviation was considered an integrated outcome of RGB-D localization, hand–eye transformation, manipulator motion, and end-effector execution, and was therefore distinguished from the independent localization accuracy of the D435-based RGB-D system.

Fruit damage observed during the harvesting experiments was classified into surface bruising, stem tearing, and cracking according to the adopted post-harvest inspection criteria (Khan et al., 2025). False-cutting cases were classified according to the deviation of the cut from the predefined acceptable stem-cutting region. These observations were used for qualitative and quantitative assessment of harvesting quality., assessed according to commercial tomato quality standards. No severe crushing or puncture damage was observed, thanks to the flexible silicone claws and controlled pneumatic shearing. The 6.6% false-cutting error refers to minor stem cuts deviating by less than 2 cm from the ideal position. In practice, these cases had negligible impact on the plants and remaining crop: clusters were still successfully harvested without dropping, and the short residual stems did not affect subsequent growth or increase disease risk in the tested tomato plants. No significant vine damage was recorded.

To further assess the long-term operational reliability of the greenhouse tomato cluster harvesting robot, accumulated continuous-operation tests were conducted under realistic greenhouse conditions. It should be emphasized that battery operating endurance and system reliability are two independent performance indicators. The battery operating endurance of the final prototype was at least 3 h per full charge. The rail-guided chassis exhibited an average station-motion response time of 0.45 ± 0.08 s and a station-positioning success rate of 97.3 ± 1.1% (Zhang et al., 2025), consistent with the use of feedback-based motion control for agricultural mobile platforms (Kang et al., 2024b).

These reliability and endurance indicators were analyzed separately from the harvesting-group comparisons and were not subjected to cross-validation. Whereas station-motion response time was treated as a continuous outcome and station-positioning success as a proportion-based outcome.

The rail-guided mobile platform autonomously travelled between predefined harvesting stations during the experiments. The proposed system achieved a system-level harvesting positioning deviation of ≤3.5 cm and maintained stable continuous operation during the greenhouse harvesting tests. The consolidated detection, maturity recognition, harvesting success, fruit-damage, and false-cutting performance of the complete system are summarized in **Table 12**. These results demonstrate the reliability of the ROS-based station-to-station motion control and multi-module coordination strategy under fixed-rail greenhouse operating conditions. Related agricultural harvesting robots have likewise been evaluated through integrated system design and field testing, highlighting the importance of whole-system validation under realistic operating conditions (Feng et al., 2026).

Representative failure cases observed during greenhouse experiments: Severe foliage occlusion occasionally reduced the visible area of the tomato cluster, resulting in missed detections or low-confidence predictions. Strong specular reflection and local overexposure could weaken maturity-related visual features and increase recognition uncertainty. In densely overlapping clusters, adjacent fruits could produce ambiguous target boundaries and reduce localization stability. RGB-D localization failures were mainly associated with invalid or unstable depth measurements in heavily occluded or reflective regions. In these cases, the corresponding target pose was rejected by the harvesting state machine and perception was repeated rather than directly triggering manipulator execution.

Whole-system harvesting failures also occurred when a valid target could not be converted into a feasible manipulator trajectory or when the end-effector failed to complete the grasping–cutting sequence. These observations indicate that the remaining limitations of the proposed system are primarily associated with extreme visual degradation, depth uncertainty, and manipulation constraints rather than with a single perception component.

The robotic harvesting experiments were conducted in the same greenhouse production area under comparable illumination and occlusion conditions. Because a single tomato cultivar was used throughout the greenhouse experiments, cultivar-related variation was not a factor in the present study. The harvesting trials were conducted across the representative maturity, illumination, and occlusion conditions described above. The whole-system harvesting samples were independent of the image dataset used for detector training and evaluation and of the samples used in the RGB-D localization experiment.

**Figure 26:**
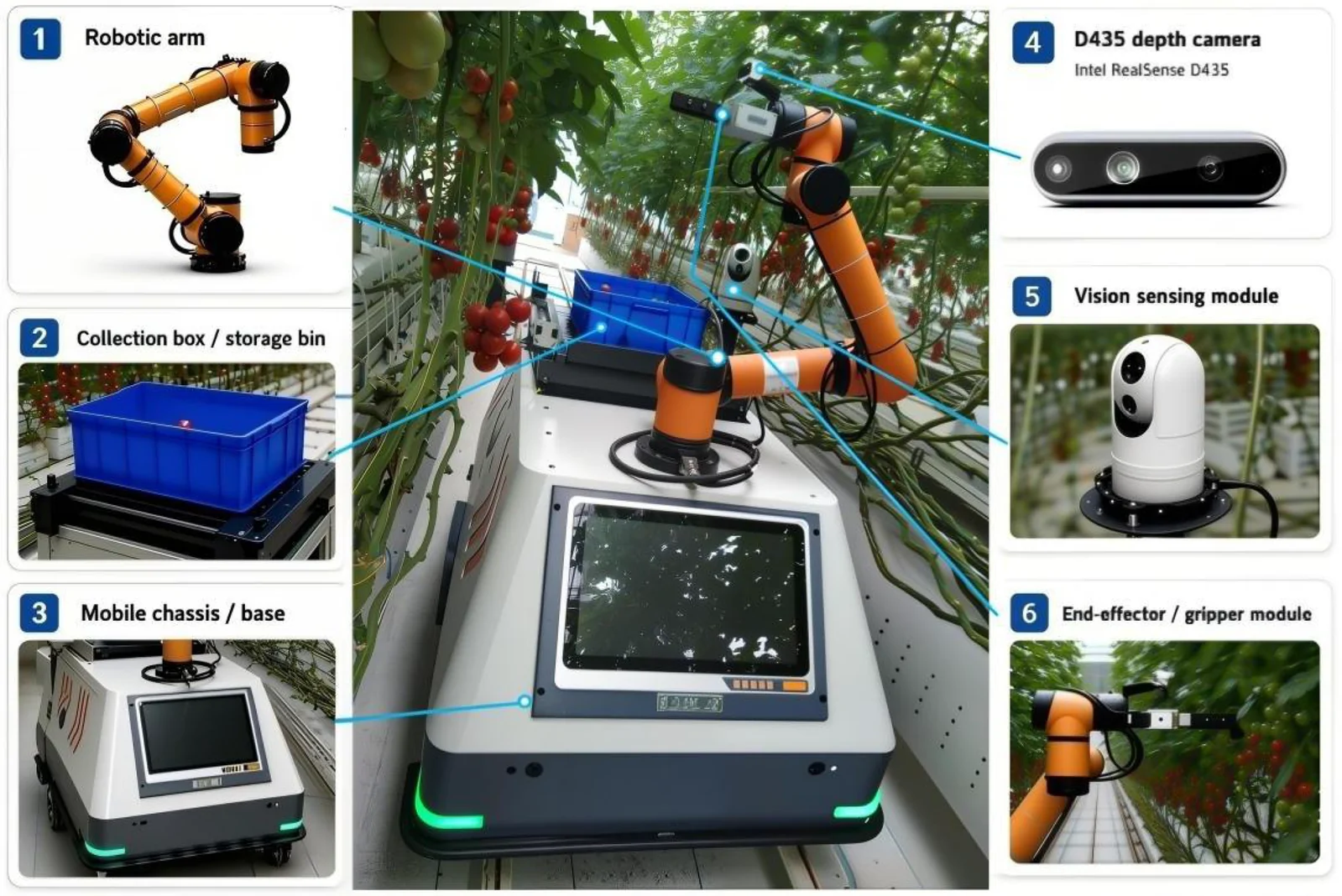
Tomato harvesting robot structure.

### 8.2 Statistical analysis

Statistical analyses were performed separately according to the type of experimental outcome. For the whole-system harvesting experiment, one tomato cluster subjected to one complete harvesting operation was defined as the statistical unit. The robotic harvesting group included 500 complete harvesting trials. Predictive-model evaluation and robotic-system evaluation were treated independently. The object-detection model was evaluated using the independent held-out test subset described in Section 8.1; cross-validation was not used to validate harvesting success, fruit damage, false cutting, mechanical positioning, or reliability-related outcomes.

For proportion-based outcomes, event counts, percentages, and 95% confidence intervals were reported when the corresponding event-count data were available. Outcomes for which the original event counts were not retained were reported descriptively as percentages without reconstructing denominators from rounded values. Wilson confidence intervals were used for binomial proportions. Effect sizes for proportion-based outcomes were expressed as absolute risk differences or relative risks with 95% confidence intervals, where the corresponding event-count data were available.

Continuous outcomes were summarized using mean ± standard deviation and 95% confidence intervals when repeated measurements were available. Comparisons were performed only when the corresponding raw observations were available and the assumptions of the selected statistical test could be assessed. Inferential comparisons were performed when the corresponding raw observations or event counts were available, whereas descriptive statistics were used to summarize the observed harvesting performance. A two-sided p-value of <0.05 was considered statistically significant.

**Table 13:** Statistical design of the principal experiments.

Outcome	Statistical unit/sample size	Reporting
Detection model	Independent test images	P,R,F1,mAP
Fruit damage	Harvested cluster	Percentage; n/N and 95% CI when event counts available
False cutting	Harvesting trial	Percentage; n/N and 95% CI when event counts available
Positioning error	Localization trial	Mean ± SD,Max

## 9 Discussion

### 9.1 Performance of the proposed detection and harvesting system

The experimental results indicate that the proposed YOLOv8n-BiFPN-WIoU framework provides an effective visual perception solution for greenhouse tomato-cluster harvesting. Compared with the baseline YOLOv8n model, the proposed configuration improved mAP@0.5 from 87.751% to 92.338% and mAP@0.5:0.95 from 65.693% to 71.368%, while also increasing Recall from 86.598% to 89.377%. The improvement in Recall is particularly relevant to robotic harvesting because missed tomato-cluster detections may directly result in unharvested targets and reduce the effective utilization of subsequent manipulation actions. RT-DETR-l was included as a representative transformer-based detector rather than as a parameter-matched lightweight competitor.

The observed improvement can be primarily attributed to the complementary effects of the BiFPN-based feature-fusion structure and WIoU-based bounding-box regression. Tomato clusters in greenhouse environments exhibit considerable variation in apparent scale, visibility, and degree of overlap. The bidirectional feature-fusion structure improves information exchange among P3, P4, and P5 feature levels, allowing shallow spatial information and deeper semantic information to be integrated more effectively. This mechanism is particularly beneficial for targets that are partially occluded or relatively small in the input image. In parallel, WIoU is introduced into the bounding-box regression branch only during training, thereby improving localization optimization without adding an additional inference-stage network branch. The combination of enhanced multi-scale representation and more robust regression provides a reasonable explanation for the improved detection performance observed in the ablation experiments. This complementary mechanism is particularly relevant to clustered tomato targets, where partial visibility affects both feature completeness and boundary localization rather than a single stage of the detection process.

The A5–A6 comparison further shows that detector selection should be aligned with the error characteristics of the downstream harvesting task. Although A6 yielded a marginally higher mAP@0.5, A5 provided higher Recall and a slightly higher mAP@0.5:0.95. Since false-negative detections directly interrupt the subsequent localization and manipulation stages, the higher Recall of A5 was considered more valuable for the integrated harvesting system, while its comparable or slightly better mAP@0.5:0.95 indicates that this choice did not compromise overall localization quality.

The edge-deployment results further indicate that the proposed detector maintained practical inference performance after conversion to an optimized TensorRT engine on the Jetson Nano platform. The measured inference speed of 21.1 FPS demonstrates that the improved detector can support near-real-time visual perception under the evaluated edge-computing conditions. Therefore, the proposed approach does not rely solely on offline detection accuracy but provides a feasible basis for integrating visual perception into a resource-constrained robotic harvesting platform. It should be noted that the object-detection metrics and whole-system maturity-recognition results characterize different stages of the harvesting process and should therefore not be directly compared.

### 9.2 Limitations and future directions

Despite the promising whole-system performance, several limitations remain. First, severe foliage occlusion, dense fruit overlap, and strong specular reflection can substantially reduce the visible target region and weaken maturity-related visual features. These conditions may lead to missed detections, reduced confidence, or ambiguous cluster boundaries. Although the proposed BiFPN-based feature fusion improves multi-scale representation, extreme visual degradation cannot always be resolved using RGB information alone.

Second, RGB-D localization remains sensitive to the quality of depth measurements. Invalid or unstable depth values may occur in heavily occluded regions, at reflective surfaces, or when the target boundary is poorly defined. In the current system, invalid target poses are rejected by the harvesting state machine and perception is repeated rather than being directly executed. This strategy improves operational safety but may also increase harvesting time when difficult targets require repeated observations.

Third, the whole-system harvesting process contains several stages in series, including detection, depth-based three-dimensional localization, hand-eye coordinate transformation, inverse kinematics, motion planning, grasping, cutting, and fruit transfer. Errors accumulated across these stages may affect the final harvesting outcome even when the visual detector performs correctly. The current system therefore still relies partly on predefined geometric relationships between the detected cluster reference point and the end-effector grasping and cutting positions. In addition, because independent mechanical bench tests of the dual-claw end-effector were not performed, failures caused by residual visual-localization errors could not be quantitatively separated from those caused by insufficient clamping retention, stem–claw slip, cutting-mechanism limitations, or mechanical wear. Future work will therefore establish a dedicated bench-test protocol covering clamping force, friction and slip, load retention, cutting reliability, and cyclic wear life.

Fourth, the rail-guided architecture improves repeatability and reduces the complexity of rail-guided station positioning, but it also limits the robot to greenhouse environments equipped with compatible fixed rails. The current system was developed primarily for station-to-station harvesting and does not address unrestricted navigation across dynamically changing greenhouse layouts. Similarly, the present prototype focuses on harvesting and collection rather than complete downstream operations such as grading, packing, or multi-robot cooperative logistics.

Future research should therefore focus on strengthening perception robustness under severe occlusion and variable illumination through multi-sensor fusion, confidence-aware depth processing, and improved temporal target validation. Closed-loop feedback between the end-effector and perception system could further reduce the influence of residual positioning errors during grasping and cutting. In addition, optimization of target selection, manipulator trajectory planning, and station positioning could improve harvesting performance beyond the current perception-level inference performance. Multi-robot cooperation and integration with subsequent conveying, grading, and collection processes may also provide a pathway toward a more comprehensive autonomous greenhouse harvesting system. Future comparative studies will also include YOLO26n and other recently released lightweight detectors under the same dataset partition, training schedule, and edge-deployment conditions.

## Data Availability

The original contributions presented in the study are included in the article/supplementary material. Further inquiries can be directed to the corresponding author.
